# Advancing Real-World Evidence Through a Federated Health Data Network (EHDEN): Descriptive Study

**DOI:** 10.2196/74119

**Published:** 2025-08-07

**Authors:** Clair Blacketer, Martijn J Schuemie, Maxim Moinat, Erica A Voss, Montse Camprubi, Peter R Rijnbeek, Patrick B Ryan

**Affiliations:** 1 OHDSI Collaborators, New York, NY, United States; 2 Department of Medical Informatics, Erasmus MC, Rotterdam, The Netherlands; 3 Johnson & Johnson (United States), 920 US Route 202, Raritan, NJ, 08869, United States, 1 7573345788; 4 Department of Biostatistics, University of California, Los Angeles, Los Angeles, CA, United States; 5 Synapse (Spain), Madrid, Spain; 6 Department of Biomedical Informatics, Columbia University, New York, NY, United States

**Keywords:** health informatics, epidemiology, electronic health records, real-world data, registries, data governance, data quality

## Abstract

**Background:**

Real-world data (RWD) are increasingly used in health research and regulatory decision-making to assess the effectiveness, safety, and value of interventions in routine care. However, the heterogeneity of European health care systems, data capture methods, coding standards, and governance structures poses challenges for generating robust and reproducible real-world evidence. The European Health Data & Evidence Network (EHDEN) was established to address these challenges by building a large-scale federated data infrastructure that harmonizes RWD across Europe.

**Objective:**

This study aims to describe the composition and characteristics of the databases harmonized within EHDEN as of September 2024. We seek to provide transparency regarding the types of RWD available and their potential to support collaborative research and regulatory use.

**Methods:**

EHDEN recruited data partners through structured open calls. Selected data partners received funding and technical support to harmonize their data to the Observational Medical Outcomes Partnership Common Data Model (OMOP CDM), with assistance from certified small-to-medium enterprises trained through the EHDEN Academy. Each data source underwent an extract-transform-load process and data quality assessment using the data quality dashboard. Metadata—including country, care setting, capture method, and population criteria—were compiled in the publicly accessible EHDEN Portal.

**Results:**

As of September 1, 2024, the EHDEN Portal includes 210 harmonized data sources from 30 countries. The highest representation comes from Italy (13%), Great Britain (12.5%), and Spain (11.5%). The mean number of persons per data source is 2,147,161, with a median of 457,664 individuals. Regarding care setting, 46.7% (n=98) of data sources reflect data exclusively from secondary care, 42.4% (n=89) from mixed care settings (both primary and secondary), and 11% (n=23) from primary care only. In terms of population inclusion criteria, 55.7% (n=117) of data sources include individuals based on health care encounters, 32.9% (n=69) through disease-specific data collection, and 11.4% (n=24) via population-based sources. Data capture methods also vary, with electronic health records (EHRs) being the most common. A total of 74.7% (n=157) of data sources use EHRs, and more than half of those (n=85) rely on EHRs as their sole method of data collection. Laboratory data are used in 29.5% (n=62) of data sources, although only one relies exclusively on laboratory data. Most laboratory-based data sources combine this method with other forms of data capture.

**Conclusions:**

EHDEN is the largest federated health data network in Europe, enabling standardized, General Data Protection Regulation–compliant analysis of RWD across diverse care settings and populations. This descriptive summary of the network’s data sources enhances transparency and supports broader efforts to scale federated research. These findings demonstrate EHDEN’s potential to enable collaborative studies and generate trusted evidence for public health and regulatory purposes.

## Introduction

Real-world data (RWD) has become a cornerstone in health care research, especially in regulatory science, due to its ability to capture insights from diverse patient populations and clinical settings. Unlike data generated through traditional randomized controlled trials, which often have stringent inclusion criteria, RWD reflects the everyday health care experiences of a broader patient base [1-5]. This breadth offers a richer context for understanding drug safety and effectiveness, guiding postauthorization safety monitoring, informing risk-benefit evaluations, and supporting regulatory decisions [6]. Regulators, industry, and academics alike rely on real-world evidence (RWE) derived from RWD to answer critical questions about health care interventions in clinical care settings that are more representative of routine practice [7-9].

Europe’s health care landscape presents both challenges and opportunities for generating RWD [10]. Its diversity spans many different health systems, terminology systems, and data collection practices, with variability in health care delivery and data availability across countries. This heterogeneity complicates large-scale representative research but also offers a unique opportunity to study diverse populations [8,11-13]. However, capturing this potential requires overcoming technical, operational, and methodological barriers to ensure data harmonization and quality. A federated network is particularly well suited to Europe’s fragmented health care landscape, where legal, linguistic, and governance diversity necessitate a model that supports local control while enabling cross-border collaboration.

Federated data networks, like the European Health Data & Evidence Network (EHDEN), are well-suited for Europe’s decentralized data landscape [14-19]. In the context of EHDEN, a federated network refers to a collaboration of independently governed data sources that retain full control of their data locally, preserving the autonomy and governance policies of individual data holders. This approach allows for multidatabase studies across diverse populations without requiring centralized data access or query execution, ensuring that personal health information does not leave its original source. By design, this method complies with the General Data Protection Regulation, as it avoids centralizing or transferring personal data and supports the principles of data minimization, purpose limitation, and local control. It is important to note that privacy-preserving practices are also in place for studies conducted using the network. Data partners (DPs) only share aggregate results, typically high-level outputs such as hazard ratios, after applying a minimum cell size threshold (k-anonymity, commonly set to 5) to suppress potentially re-identifiable results.

EHDEN was established as an Innovative Medicines Initiative (IMI), now Innovative Health Initiative, public-private partnership in November 2018 to overcome the challenges and transform how health data is used in Europe [20,21]. The project built a federated data network that standardizes health data across participating sources, making data analysis more feasible and consistent. By harmonizing data and implementing quality assurance protocols, EHDEN enhances the usability and comparability of RWD across Europe. This paper provides an overview of the EHDEN network, examining its data harmonization efforts, quality control processes, and the range of data sources included in the network. Through this discussion, we aim to highlight the scope of RWD available across Europe and its potential for advancing health care research and regulatory decision-making.

## Methods

### Common Data Model

As the foundation for its network, EHDEN adopted the Observational Medical Outcomes Partnership Common Data Model (OMOP CDM) [22-24]. The OMOP CDM is widely recognized for its “structure + content” approach whereby the tables and fields (structure) as well as the vocabulary (content) are standardized, allowing for integration of data across multiple systems while maintaining data integrity. The model also supports a wide range of data types, including electronic health records (EHRs), claims data, and patient registries.

The OMOP CDM is maintained by the Observational Health Data Science and Informatics (OHDSI) community, an open science effort that aims to improve health by empowering a community to collaboratively generate the evidence that promotes better health decisions and better care [25]. The open-source nature of OHDSI allows for continuous community-driven improvements, making it adaptable to emerging health care needs [26-28].

### Data Partner Calls

**Figure 1.**
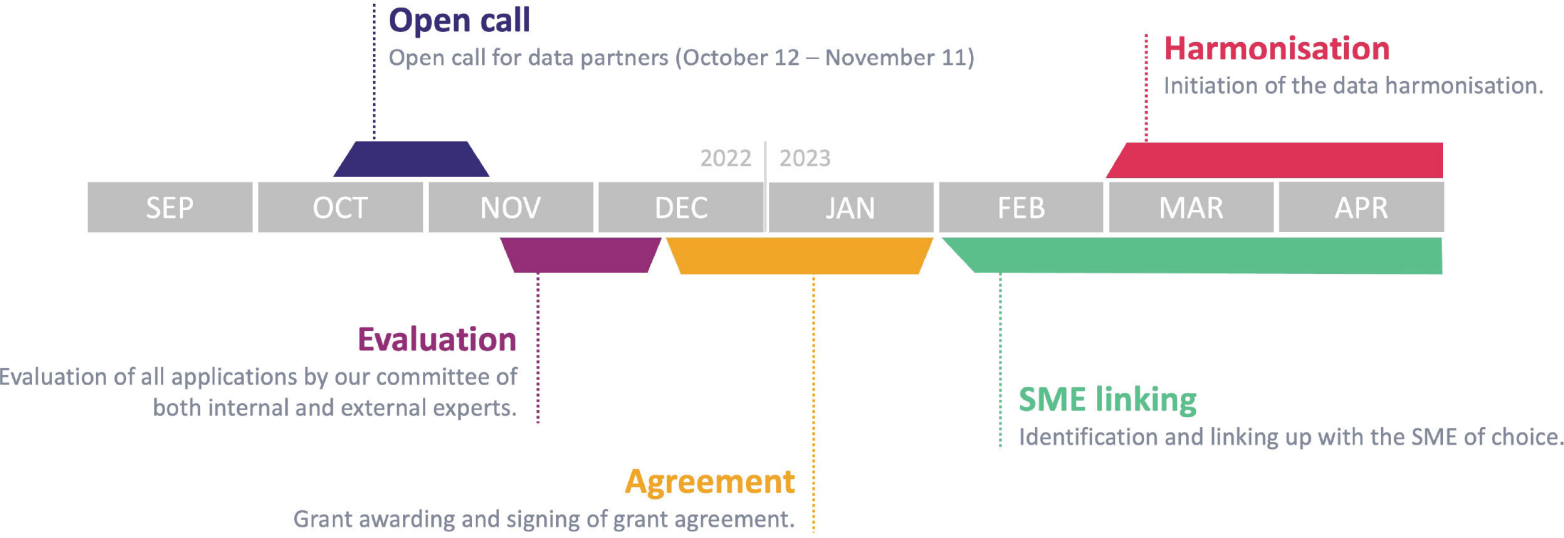
Timeline of data call 7 (2022‐2023) within the European Health Data & Evidence Network. The figure illustrates key milestones in the selection, contracting, and data harmonization process for data partners onboarded during this call. SME: small-to-medium enterprise﻿

Any organization with access to a data source in Europe could apply to be included in the EHDEN network. In this context, a data source is defined as a distinct repository of health care-related data pertaining to a specific set of individuals. Except for the COVID-19 Rapid Collaboration Call, the 7 DP calls executed between September 2019 and October 2022 were aligned to similar timelines for DP identification, grant awarding, and initiation of data harmonization (Figure 1). In each call, candidate partner organizations with access to one or more electronic health care databases applied to the EHDEN Harmonization Fund for a grant to implement or enhance their database (Multimedia Appendix 1). DPs were selected based on 3 criteria: data impact (size, coverage, quality), network impact (track record, uniqueness within network), and readiness (willingness to participate, governance) (Multimedia Appendix 2), reviewed by a Data Source Prioritization Committee. Each application was reviewed and scored by 2 reviewers, and the top applicants per round were awarded a grant.

### Data Standardization

Once DPs were identified and grants awarded, each data source underwent standardization to the OMOP CDM. A crucial factor in EHDEN’s long-term sustainability and success was the recruitment and training of local small-to-medium enterprises (SMEs). These SMEs were brought on board through separate calls from the EHDEN consortium and certified via the EHDEN Academy education program concluded by an onsite or web-based training. SMEs played a pivotal role in supporting DPs throughout the extract, transform, and load (ETL) process by providing guidance and expertise. In total, 64 SMEs across 22 countries were certified by EHDEN to support DPs.

The ETL process followed by the DPs and supported by the SMEs was largely uniform, as outlined by Voss et al [24], and involved four key steps: (1) summarizing the native data, (2) creating the ETL specification, (3) mapping source vocabulary codes, and (4) implementing the ETL. This standardized approach ensured transparency in the followed procedure and adherence to the conventions in converting data sources to the CDM, while also allowing DPs to benefit from the SMEs’ specialized knowledge.

To promote semantic interoperability across heterogeneous health care systems, all source codes for diagnoses, medications, procedures, and measurements were mapped to standardized vocabularies (eg, SNOMED CT [Systematized Nomenclature of Medicine – Clinical Terms], RxNorm [Prescription Normalized Names], LOINC [Logical Observation Identifiers Names and Codes]) as required by the OMOP CDM. For example, the UK Biobank contributed data that included SNOMED CT-coded diagnoses from EHRs alongside custom-coded fields for self-reported conditions and blood pressure measurements. Mapping these nonstandard elements involved a combination of automated matching and manual curation followed by expert validation. This process facilitated consistent interpretation of clinical concepts across countries and enhanced the analytical interoperability of the network [29].

Payments were structured based on output; to receive full funding, DPs were required to meet 3 different milestones. The ETL specification document entitled DPs to 30%, ETL implementation and infrastructure released the next 40%, and the final 30% was received by the DP after final inspection of the harmonized data (Multimedia Appendix 3).

### Data Quality

Each milestone was reviewed by an EHDEN consortium member who was part of the Milestone review committee. The ETL specification document required by milestone 1 was evaluated to ensure the mapping adhered to the OMOP CDM conventions and that the DPs or SMEs had a good understanding of the CDM and their own native data [23,30]. Milestone 2, the ETL implementation, had multiple review steps. The infrastructure was investigated to be sure the DPs were using a supported database platform [31]. The vocabulary mapping was evaluated to ensure most, if not all, source codes were included. The data quality dashboard (DQD) was developed by EHDEN Work Package 5 to provide a standard structure for quality assurance [32]. It was used by DPs throughout the ETL process to continually improve the standardized data sources [33]. As described by Voss et al [24], the median number of times a DP ran the DQD was 3 (IQR 2‐7). In general, conformance issues to the OMOP CDM were identified and addressed in initial runs, with more complex site-specific vocabulary mapping issues addressed in subsequent runs. DPs used the default failure thresholds in the first run. These were updated to reflect the nuances of each data source in later runs of the software. In milestone 3, final DQD results as well as the CDM Inspection Report were reviewed [34]. Once approved, the DP then entered their information into the EHDEN portal, an online platform open to the public designed to catalog metadata on each data source [35].

### Analyses

An individual data source was considered one entry in the EHDEN portal. Data sources were categorized based on country, person count, the levels of care represented (primary, secondary, or mixed), why a person was included, and how data were captured. These categories were ascertained from the data source description and metadata provided to the EHDEN portal and verified with the DPs.

There are 3 reasons persons could be included in an EHDEN data source (person inclusion), as defined by population, where a person enters the data source because they live in a certain geographical location or because they are registered with a practice or insurer; encounter, where a person enters the data source upon a visit to a health care provider for any medical reason; or disease, where a person enters the data source when satisfying specific criteria (ie, a person has a specific medical condition). The most restrictive reason was chosen as the classification for each data source.

We identified which types of data capture methods each source contained; it could be one or more of the following: EHR, a bill or adjudicated claim record for health services rendered (claim), measurements taken and results recorded (laboratory), a set of required information collected about participants in a registry (case report form), patient-reported data (survey), documents analyzed by pulling structured data from unstructured data using a natural language processing algorithm, or death information from an official source or government entity (death certificate). If the data source did not provide this information, they were categorized as unknown.

### Ethical Considerations

Patients or the public were not involved in the design, conduct, reporting, or dissemination plans of this research.

## Results

**Figure 2.**
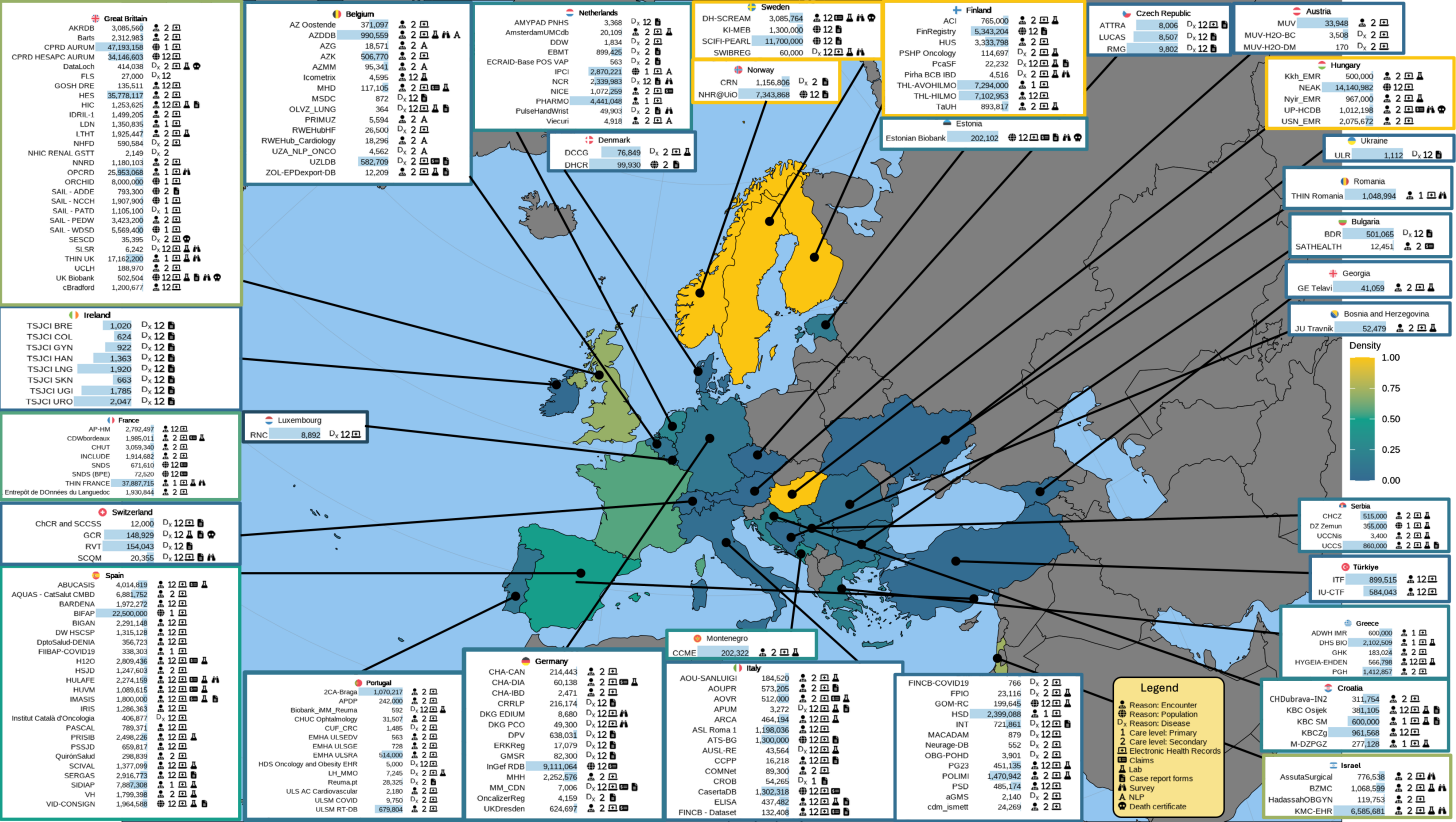
Geographic distribution and characteristics of data sources included in the European Health Data & Evidence Network as of September 1, 2024. The map displays country-level data density based on the number of data sources relative to national population size. Overlaid symbols represent key metadata for each source, including total person count, care setting (primary, secondary, or mixed), data capture methods (eg, electronic health records, laboratory, or claims), and the reason for person inclusion (eg, encounter-based, disease-specific, or population-based).

As of September 1, 2024, there are 210 data sources in the EHDEN portal. Figure 2 shows all countries and the number of data sources available in each. The data sources span 30 countries, with the largest representation from Italy, Great Britain, and Spain, with 13%, 12.5%, and 11.5% of the total data sources in the network, respectively. The mean number of persons per data source is 2,147,161 and the median number of persons is 457,664.

Table 1 provides the complete list of data sources and their attributes. One row in the table equates to 1 data source. The first column lists the DP, which is the name of the institution or organization that is the custodian of the data source. The individual data sources are identified by an acronym, which is also how they are identified in the EHDEN portal. Country of origin is represented by the 2-digit country code. The number of persons, the person inclusion method, and care level are also provided. Each data capture category has its own column in the table. If a data source uses one of the capture methods, that box is filled with a check mark symbol in the table.

**Table 1.** Overview of 210 standardized real-world data sources in the European Health Data & Evidence Network as of September 1, 2024. The table includes the full list of data sources by country, total number of persons represented, person inclusion, care level, and data capture methods. These attributes provide essential context for understanding the scope and scale of data available for real-world evidence generation within the European Health Data & Evidence Network.

Data partner	Data source acronym	Country	Person count	Person inclusion	Care level	EHR^a^	Claim	Lab	Case report form	Survey	NLP^b^	Death certificate	Unknown
Centro Clínico Académico – Braga, Associação (2CA-Braga)	2CA-Braga	Portugal	10,70,217	Encounter	Secondary	✓							
INCLIVA	ABUCASIS	Spain	40,14,819	Encounter	Mixed	✓	✓	✓					
The wellbeing services county of Southwest Finland, VarHa	ACI	Finland	7,65,000	Encounter	Secondary	✓		✓					
Innovative Medical Research SA	ADWH IMR	Greece	6,00,000	Encounter	Primary	✓							
Fondazione Casa Sollievo della Sofferenza	aGMS	Italy	2140	Disease	Secondary	✓							
Akrivia Health	AKRDB	Great Britain	30,85,560	Encounter	Secondary	✓							
Amsterdam UMC	AmsterdamUMCdb	Netherlands	20,109	Encounter	Secondary	✓		✓					
Stichting VUmc	AMYPAD PNHS	Netherlands	3368	Disease	Mixed				✓				
AZIENDA OSPEDALIERO UNIVERSITARIA SAN LUIGI GONZAGA	AOU-SANLUIGI	Italy	1,84,520	Encounter	Secondary	✓		✓					
University Hospital of Parma	AOUPR	Italy	5,73,205	Encounter	Secondary	✓			✓				
Azienda Ospedaliera Universitaria Integrata Verona	AOVR	Italy	5,12,000	Encounter	Secondary	✓	✓	✓					
Assistance Publique - Hopitaux de Marseille	AP-HM	France	27,92,497	Encounter	Mixed	✓							
APDP	APDP	Portugal	2,42,000	Encounter	Secondary	✓							
Azienda Ospedaliero-Universitaria di Modena	APUM	Italy	3272	Disease	Mixed	✓		✓	✓				
Servei Català de la Salut	AQUAS - CatSalut CMBD	Spain	68,81,752	Encounter	Secondary	✓							
FONDAZIONE TOSCANA GABRIELE MONASTERIO PER LA RICERCA MEDICA E DI SANITA PUBBLICA (FTGM)	ARCA	Italy	4,64,194	Encounter	Mixed	✓		✓					
ASL Roma 1	ASL Roma 1	Italy	11,98,036	Encounter	Mixed	✓							
Assuta medical centers	AssutaSurgical	Israel	7,76,538	Encounter	Secondary	✓				✓			
ATS Bergamo	ATS-BG	Italy	13,00,000	Population	Mixed	✓			✓				
Institute of Rheumatology	ATTRA	Czech Republic	8006	Disease	Mixed	✓			✓				
Marco Massari (IRCSSE)	AUSL-RE	Italy	43,564	Disease	Mixed	✓		✓					
Az Oostende	AZ Oostende	Belgium	3,71,097	Encounter	Secondary	✓							
AZ Delta	AZDDB	Belgium	9,90,559	Encounter	Secondary	✓		✓		✓	✓		
VZW AZ Groeninge	AZG	Belgium	18,571	Encounter	Secondary	✓					✓		
AZ Klina	AZK	Belgium	5,06,770	Encounter	Secondary	✓							
AZ Maria Middelares	AZMM	Belgium	95,341	Encounter	Secondary						✓		
Servicio Navarro de Salud Osasunbidea (SNS-O)	BARDENA	Spain	19,72,272	Encounter	Mixed	✓							
Barts Health NHS Trust	Barts	Great Britain	23,12,983	Encounter	Secondary	✓							
National Scientific Program “E-Health in Bulgaria”	BDR	Bulgaria	5,01,065	Disease	Mixed	✓			✓				
Agencia Española de Medicamentos y Productos Sanitarios, AEMPS	BIFAP	Spain	########	Population	Primary	✓							
Instituto Aragonés de Ciencias de la Salud (IACS)	BIGAN	Spain	22,91,148	Encounter	Mixed	✓							
Instituto de Medicina Molecular	Biobank_iMM_Reuma	Portugal	592	Disease	Mixed	✓		✓					
Bnai Zion Medical Research Foundation and Infrastructure Development Health Services	BZMC	Israel	10,68,599	Encounter	Secondary	✓		✓		✓			
Inspire-srl	CasertaDB	Italy	13,02,318	Population	Mixed	✓	✓						
Connected Bradford	cBradford	Great Britain	12,00,677	Encounter	Mixed	✓							
Clinical Center of Montenegro	CCME	Montenegro	2,02,322	Encounter	Secondary	✓		✓					
Casa di Cura Privata del Policlinico (CCPP)	CCPP	Italy	16,218	Encounter	Mixed	✓			✓				
ISMETT	cdm_ismett	Italy	24,269	Encounter	Secondary	✓							
Bordeaux University Hospital	CDWbordeaux	France	19,85,011	Encounter	Secondary	✓	✓	✓					
Charité - Universitätsmedizin	CHA-CAN	Germany	2,14,443	Encounter	Secondary	✓							
Charité - Universitätsmedizin	CHA-DIA	Germany	60,138	Encounter	Secondary	✓	✓	✓					
Charité - Universitätsmedizin	CHA-IBD	Germany	2471	Encounter	Secondary	✓							
Institute of Social and Preventive Medicine, University of Bern	ChCR and SCCSS	Switzerland	12,000	Disease	Mixed	✓			✓				
Clinical-hospital center Zvezdara	CHCZ	Serbia	5,15,000	Encounter	Secondary	✓		✓					
Clinical Hospital Dubrava	CHDubrava–IN2	Croatia	3,11,754	Encounter	Secondary	✓							
Centro Hospitalar Universitário de Coimbra (CHUC)	CHUC Ophtalmology	Portugal	31,507	Encounter	Secondary	✓							
Center Hospitalier Universitaire de Toulouse	CHUT	France	30,59,340	Encounter	Secondary	✓							
Modena Oncology Center - Azienda Ospedaliera Modena	COMNet	Italy	89,300	Encounter	Secondary	✓							
Clinical Practice Research Datalink (CPRD)	CPRD AURUM	Great Britain	########	Population	Primary	✓							
Clinical Practice Research Datalink (CPRD)	CPRD HESAPC AURUM	Great Britain	########	Population	Mixed	✓							
The Norwegian Cancer Registry	CRN	Norway	11,56,806	Disease	Secondary	✓			✓				
Basilicata Cancer Registry	CROB	Italy	54,265	Disease	Primary				✓				
Krebsregister Rheinland-Pfalz	CRRLP	Germany	2,16,174	Disease	Mixed				✓				
CUF	CUF_CRC	Portugal	1485	Disease	Secondary	✓							
DataLoch	DataLoch	Great Britain	4,14,038	Disease	Secondary	✓		✓				✓	
Center for Surgical Science (CSS)	DCCG	Denmark	76,849	Disease	Secondary	✓							
Amsterdam UMC	DDW	Netherlands	1834	Disease	Secondary	✓							
Stockholm CREAtinine Measurements Project	DH-SCREAM	Sweden	30,85,764	Encounter	Mixed		✓	✓	✓	✓		✓	
University of Southern Denmark	DHCR	Denmark	99,930	Population	Secondary								
DIGITAL HEALTH SOLUTIONS SA	DHS BIO	Greece	21,02,509	Encounter	Primary	✓		✓					
German Cancer Society (DKG)	DKG EDIUM	Germany	8680	Disease	Mixed	✓				✓			
German Cancer Society (DKG)	DKG PCO	Germany	49,300	Disease	Mixed	✓				✓			
Hospital de Denia	DptoSalud-DENIA	Spain	3,56,723	Encounter	Mixed	✓							
University of Ulm, ZIBMT	DPV	Germany	6,38,031	Disease	Mixed				✓				
Research Institute - Hospital de la Santa Creu i Sant Pau	DW HSCSP	Spain	13,15,128	Encounter	Mixed	✓		✓					
Primary Healthcare Center Zemun	DZ Zemun	Serbia	3,55,000	Population	Primary	✓		✓					
EBMT: The European Society for Blood and Marrow Transplantation	EBMT	Netherlands	8,99,425	Disease	Secondary				✓				
European Clinical Research Alliance on Infectious Diseases (ECRAID)	ECRAID-Base POS VAP	Netherlands	563	Disease	Secondary				✓				
Center Hospitalier Universitaire de Montpellier	eDOL Entrepôt de DOnnées du Languedoc	France	19,30,844	Encounter	Secondary	✓							
Fondazione IRCCS Policlinico San Matteo	ELISA	Italy	4,37,482	Encounter	Mixed	✓		✓	✓				
EGAS MONIZ HEALTH ALLIANCE	EMHA ULSEDV	Portugal	563	Encounter	Secondary	✓							
EGAS MONIZ HEALTH ALLIANCE	EMHA ULSGE	Portugal	728	Encounter	Secondary	✓							
EGAS MONIZ HEALTH ALLIANCE	EMHA ULSRA	Portugal	5,14,000	Encounter	Secondary	✓							
European Rare Kidney Disease Registry (ERKReg)	ERKReg	Germany	17,079	Disease	Mixed				✓				
University of Tartu	Estonian Biobank	Estonia	2,02,102	Population	Mixed	✓	✓		✓	✓		✓	
FIIBAP	FIIBAP-COVID19	Spain	3,38,303	Encounter	Primary	✓							
Fondazione IRCCS Istituto Neurologico Carlo Besta	FINCB - Dataset	Italy	1,32,408	Encounter	Mixed	✓	✓		✓				
Fondazione IRCCS Istituto Neurologico Carlo Besta FINCB	FINCB-COVID19	Italy	766	Disease	Secondary	✓							
FinRegistry (Institute of Molecular Medicine Finland (FIMM), University of Helsinki)	FinRegistry	Finland	53,43,204	Population	Mixed				✓				
Queen Mary University of London	FLS	Great Britain	27,000	Disease	Mixed								✓
Fondazione Poliambulanza Istituto Ospedaliero	FPIO	Italy	23,116	Disease	Secondary	✓		✓					
Geneva Cancer Registry	GCR	Switzerland	1,48,929	Disease	Mixed			✓	✓			✓	
Telavi Regional Hospital	GE Telavi	Georgia	41,059	Encounter	Secondary	✓		✓					
GENERAL HOSPITAL OF KAVALA	GHK	Greece	1,83,024	Encounter	Secondary	✓							
MS Forschungs- und Projektentwicklungs-GmbH	GMSR	Germany	82,300	Disease	Mixed	✓			✓				
Grande Ospedale Metropolitano “Bianchi-Melacrino-Morelli”	GOM-RC	Italy	1,99,645	Population	Mixed	✓		✓					
GOSH	GOSH DRE	Great Britain	1,35,511	Encounter	Mixed	✓							
Fundacion de Investigacion Biomedica del Hospital Universitario 12 de Octubre	H12O	Spain	28,09,436	Encounter	Mixed	✓	✓	✓					
Hadassah OBGYN	HadassahOBGYN	Israel	1,19,753	Encounter	Secondary	✓							
Hospital Distrital de Santarém (HDS)	HDS Oncology and Obesity EHR	Portugal	5000	Disease	Mixed	✓							
Harvey Walsh Ltd	HES	Great Britain	########	Encounter	Secondary	✓							
Health Informatics Center (HIC)	HIC	Great Britain	12,53,625	Encounter	Mixed	✓		✓	✓				
SIMG	HSD	Italy	23,99,088	Encounter	Primary	✓							
Hospital Sant Joan de Déu	HSJD	Spain	12,47,603	Encounter	Secondary	✓							
Fundación para la Investigación del Hospital Universitario La Fe de la Comunidad Valenciana (HULAFE)	HULAFE	Spain	22,74,159	Encounter	Mixed	✓	✓	✓		✓			
Hospital District of Helsinki and Uusimaa	HUS	Finland	33,33,798	Encounter	Secondary	✓							
Virgen Macarena University Hospital	HUVM	Spain	10,89,615	Encounter	Mixed	✓	✓	✓					
DIAGNOSTIC & THERAPEUTIC CENTER OF ATHENS “HYGEIA” SINGLE MEMBER SOCIETE ANONYME	HYGEIA-EHDEN	Greece	5,66,798	Encounter	Mixed	✓		✓					
Icometrix	Icometrix	Belgium	4595	Encounter	Mixed			✓					
Lancashire and South Cumbria Integrated Care Board	IDRIL-1	Great Britain	14,99,205	Encounter	Secondary	✓							
Fundacio Institut d’Investigacions Mèdiques (FIMIM)	IMASIS	Spain	18,00,000	Encounter	Mixed	✓	✓	✓	✓				
Lille University Hospital	INCLUDE	France	1,914.68	Encounter	Secondary	✓							
InGef - Institute for Applied Health Research Berlin GmbH	InGef RDB	Germany	91,11,064	Population	Mixed		✓						
Institut Català d’Oncologia	Institut Català d’Oncologia	Spain	4,06,877	Disease	Mixed	✓							
Fondazione Istituto Nazionale dei Tumori	INT	Italy	7,21,861	Disease	Mixed	✓			✓				
NO GRANT	IPCI	Netherlands	28,70,221	Population	Primary	✓		✓			✓		
Consorci Corporació Sanitària Parc Taulí	IRIS	Spain	12,86,363	Encounter	Mixed	✓							
Istanbul University	ITF	Turkey	8,99,515	Encounter	Mixed	✓							
IUC Cerrahpaşa TIP Fakületesi	IU-CTF	Turkey	5,84,043	Encounter	Mixed	✓							
E-MEDIT D.O.O. & Hospital Travnik	JU Travnik	Bosnia and Herzegovina	52,479	Encounter	Secondary	✓		✓					
IN2 d.o.o. & Clinical Hospital Center Osijek	KBC Osijek	Croatia	3,81,105	Encounter	Mixed	✓		✓	✓				
IGEA d.o.o. & University Hospital Center Sestre milosrdnice	KBC SM	Croatia	6,00,000	Encounter	Primary	✓		✓	✓				
Hierarchia & University Hospital Center Zagreb	KBCZg	Croatia	9,61,568	Encounter	Mixed	✓							
MEB KI	KI-MEB	Sweden	13,00,000	Population	Mixed				✓				
Bács-Kiskun Megyei Kórház a Szegedi Tudományegyetem Általános Orvostudományi Kar Oktató Kórháza	Kkh_EMR	Hungary	5,00,000	Encounter	Secondary	✓		✓					
The Directorate of Government Medical Centers at the Israeli Ministry Of Health	KMC-EHR	Israel	65,85,681	Encounter	Secondary	✓		✓		✓			
Lambeth DataNet	LDN	Great Britain	13,50,835	Encounter	Primary								
Hospital da Luz Learning Health	LH_MMO	Portugal	7245	Disease	Secondary	✓	✓	✓				✓	
Leeds Teaching Hospitals	LTHT	Great Britain	19,25,447	Encounter	Secondary	✓		✓					
OAKS Consulting s.r.o.	LUCAS	Czech Republic	8507	Disease	Mixed				✓				
MCS Grupa d.o.o. & Health Care Center of Primorje-Gorski Kotar County	M-DZPGZ	Croatia	2,77,128	Encounter	Primary	✓		✓					
Azienda Ospedaliera SS Antonio e Biagio e Cesare Arrigo	MACADAM	Italy	879	Disease	Mixed	✓							
Medaman	MHD	Belgium	1,17,105	Encounter	Secondary	✓	✓	✓					
Hanover Medical School	MHH	Germany	22,52,576	Encounter	Secondary	✓							
CancerDataNet GmbH	MM_CDN	Germany	7006	Disease	Mixed	✓	✓		✓				
University MS Center	MSDC	Belgium	872	Disease	Mixed				✓				
Medical University of Vienna	MUV	Austria	33,948	Encounter	Secondary	✓							
Medical University of Vienna	MUV-H2O-BC	Austria	3508	Disease	Secondary	✓							
Medical University of Vienna	MUV-H2O-DM	Austria	170	Disease	Secondary	✓							
IKNL	NCR	Netherlands	23,39,983	Disease	Mixed			✓	✓	✓			
National Institute of Health Insurance Fund Management Hungary	NEAK	Hungary	14,140,982	Population	Mixed	✓							
AO Card. G. Panico - Center for Neurodegenerative Diseases and Aging Brain	Neurage-DB	Italy	552	Disease	Secondary	✓							
Queen Mary University of London	NHFD	Great Britain	5,90,584	Disease	Secondary	✓							
King’s College London	NHIC RENAL GSTT	Great Britain	2149	Disease	Secondary			✓					✓
University of Oslo	NHR@UiO	Norway	73,43,868	Population	Mixed				✓				
National Intensive Care Evaluation foundation	NICE	Netherlands	10,72,259	Encounter	Secondary	✓	✓						
UK National Neonatal Research Database	NNRD	Great Britain	11,80,103	Encounter	Secondary	✓							
Szabolcs-Szatmár-Bereg Megyei Kórházak és Egyetemi Oktatókórház	Nyir_EMR	Hungary	9,67,000	Encounter	Secondary	✓		✓					
Bambino Gesù Children’s Hospital	OBG-POHD	Italy	3901	Disease	Secondary	✓							
Onze-Lieve-Vrouwziekenhuis Aalst-Asse-Ninove	OLVZ_LUNG	Belgium	364	Disease	Mixed	✓		✓	✓				
GermanOncology	OncalizerReg	Germany	4159	Disease	Secondary				✓				
Optimum Patient Care Limited	OPCRD	Great Britain	25,953,068	Encounter	Primary	✓				✓			
Royal College of General Practitioners (RCGP)	ORCHID	Great Britain	80,00,000	Population	Primary	✓							
Rioja Salud	PASCAL	Spain	7,89,371	Encounter	Mixed	✓							
University of Turku (Prostate Cancer Registry of South West Finland)	PcaSF	Finland	22,232	Disease	Mixed	✓		✓	✓				
ASST Papa Giovanni XXIII	PG23	Italy	4,51,135	Encounter	Mixed	✓		✓					
Papageorgiou General Hospital	PGH	Greece	14,12,857	Encounter	Secondary	✓							
STIZON	PHARMO	Netherlands	44,41,048	Encounter	Primary	✓							
BCB Medical Ltd	Pirha BCB IBD	Finland	4516	Disease	Secondary	✓		✓		✓			
Fondazione IRCCS Ca’ Granda Ospedale Maggiore Policlinico	POLIMI	Italy	14,70,942	Encounter	Secondary	✓		✓					
UZ Brussel	PRIMUZ	Belgium	5594	Encounter	Secondary			✓			✓		
Fundació Institut d´Investigació Sanitària Illes Balears	PRISIB	Spain	24,98,226	Encounter	Mixed	✓		✓					
IRCCS Policlinico San Donato	PSD	Italy	4,85,174	Encounter	Mixed	✓		✓					
Finnish Clinical Biobank Tampere	PSHP Oncology	Finland	1,14,697	Disease	Secondary	✓		✓					
Parc Sanitari Sant Joan de Déu	PSSJD	Spain	6,59,817	Encounter	Mixed	✓							
Harm Slijper	PulseHandWrist	Netherlands	49,903	Disease	Secondary				✓	✓			
Quironsalud	QuirónSalud	Spain	2,98,839	Encounter	Secondary	✓							
Registo Portugues de Doentes Reumaticos	Reuma.pt	Portugal	28,325	Disease	Secondary				✓				
Czech Myeloma Group	RMG	Czech Republic	9802	Disease	Mixed				✓				
Registre National du Cancer du Luxembourg	RNC	Luxembourg	8892	Disease	Mixed	✓							
Vaud Cancer Registry	RVT	Switzerland	1,54,043	Disease	Mixed				✓				
LynxCare	RWEHub_Cardiology	Belgium	18,296	Encounter	Secondary						✓		
LynxCare	RWEHubHF	Belgium	26,500	Disease	Secondary	✓							
SAIL Databank	SAIL - ADDE	Great Britain	7,93,300	Population	Secondary				✓				
SAIL Databank	SAIL - NCCH	Great Britain	19,07,900	Population	Primary	✓							
SAIL Databank	SAIL - PATD	Great Britain	11,05,100	Disease	Primary	✓							
SAIL Databank	SAIL - PEDW	Great Britain	34,23,200	Encounter	Secondary	✓							
SAIL Databank	SAIL - WDSD	Great Britain	55,69,400	Population	Primary	✓		✓				✓	
SAT Health	SATHEALTH	Bulgaria	12,451	Encounter	Secondary		✓						
Gothenburg University	SCIFI-PEARL	Sweden	11,700,000	Population	Mixed			✓	✓				
Servicio Cántabro de Salud and IDIVAL	SCIVAL	Spain	13,77,099	Encounter	Mixed	✓		✓					
HUG and SCQM	SCQM	Switzerland	20,355	Disease	Mixed	✓			✓	✓			
Consellería de Sanidade	SERGAS	Spain	29,16,773	Encounter	Mixed	✓			✓				
University of Edinburgh	SESCD	Great Britain	35,395	Disease	Secondary	✓						✓	
SIDIAP - The Information System for Research in Primary Care	SIDIAP	Spain	78,87,308	Encounter	Primary	✓		✓					
King’s College London	SLSR	Great Britain	6242	Disease	Mixed	✓		✓		✓			
Health Data Hub	SNDS	France	671,610^c^	Population	Mixed		✓						
Bordeaux PharmacoEpi	SNDS (BPE)	France	72,520^c^	Population	Mixed		✓						
SWIBREG	SWIBREG	Sweden	60,000	Disease	Mixed	✓		✓		✓			
Pirkanmaa Hospital District	TaUH	Finland	8,93,817	Encounter	Secondary	✓		✓					
CEGEDIM HEALTH DATA	THIN FRANCE	France	########	Encounter	Primary	✓		✓		✓			
CEGEDIM HEALTH DATA	THIN Romania	Romania	10,48,994	Encounter	Primary	✓				✓			
CEGEDIM HEALTH DATA	THIN UK	Great Britain	########	Encounter	Primary	✓		✓		✓			
Finnish Institute of Health and Welfare	THL-AVOHILMO	Finland	72,94,000	Encounter	Primary	✓							
Finnish Institute for Health and Welfare (THL)	THL-HILMO	Finland	71,02,953	Encounter	Mixed	✓							
Trinity St James’s Cancer Institute	TSJCI BRE	Ireland	1020	Disease	Mixed				✓				
Trinity St James’s Cancer Institute	TSJCI COL	Ireland	624	Disease	Mixed				✓				
Trinity St James’s Cancer Institute	TSJCI GYN	Ireland	922	Disease	Mixed				✓				
Trinity St James’s Cancer Institute	TSJCI HAN	Ireland	1363	Disease	Mixed				✓				
Trinity St James’s Cancer Institute	TSJCI LNG	Ireland	1920	Disease	Mixed				✓				
Trinity St James’s Cancer Institute	TSJCI SKN	Ireland	663	Disease	Mixed				✓				
Trinity St James’s Cancer Institute	TSJCI UGI	Ireland	1785	Disease	Mixed				✓				
Trinity St James’s Cancer Institute	TSJCI URO	Ireland	2047	Disease	Mixed				✓				
Clinical center of Nis	UCCNis	Serbia	3400	Encounter	Secondary	✓		✓					
Clinical Center of Serbia	UCCS	Serbia	8,60,000	Encounter	Secondary	✓		✓	✓				
University College London Hospitals	UCLH	Great Britain	1,88,970	Encounter	Secondary	✓							
University College London (UCL) (UK Biobank)	UK Biobank	Great Britain	5,02,504	Population	Mixed	✓		✓	✓	✓		✓	
University Medicine Dresden	UKDresden	Germany	6,24,697	Encounter	Secondary	✓	✓						
National Cancer Institute	ULR	Ukraine	1112	Disease	Mixed				✓				
ULS AC Cardiovascular	ULS AC Cardiovascular	Portugal	2180	Encounter	Secondary	✓							
ULSM	ULSM COVID	Portugal	9750	Disease	Secondary	✓							
Unidade Local de Saúde de Matosinhos	ULSM RT-DB	Portugal	6,79,804	Encounter	Secondary	✓							
University of Pécs	UP-HCDB	Hungary	10,12,198	Encounter	Secondary	✓	✓			✓		✓	
Semmelweis University	USN_EMR	Hungary	20,75,672	Encounter	Secondary	✓							
University Hospital Antwerp	UZA_NLP_ONCO	Belgium	4562	Disease	Secondary						✓		
Universitaire Ziekenhuizen KU Leuven	UZLDB	Belgium	5,82,709	Disease	Secondary	✓	✓		✓				
Vall d’Hebrón Hospital Campus	VH	Spain	17,99,398	Encounter	Secondary	✓		✓					
FISABIO-HSRU	VID-CONSIGN	Spain	19,64,588	Population	Mixed	✓		✓	✓				
VieCuri Medisch Centrum	Viecuri	Netherlands	4918	Encounter	Secondary	✓					✓		
Ziekenhuis Oost-Limburg	ZOL-EPDexport-DB	Belgium	12,209	Encounter	Secondary	✓		✓	✓				

a EHR: electronic health record.

b NLP: natural language processing.

c This is a subset of the full data source.

Looking at care settings, 46.7% (98/210) of data sources represent data from the secondary setting only, while 42.4% (89/210) represent data from mixed settings (primary and secondary). A comparatively smaller set of 11.0% (23/210) represents data only from the primary care setting (Table 2). Looking at the ways in which persons are included in the data sources, 55.7% (n=117) do so through health care encounters, 32.9% (n=69) through disease-specific data collection, and 11.4% (n=24) through population-based sources.

**Table 2.** Stratification of data sources in the European Health Data & Evidence Network by method of person inclusion and care level. Person inclusion reflects the basis by which individuals are represented in the database: health care encounters, disease-specific inclusion, or population-based inclusion. Care settings indicate whether data were captured in primary care, secondary (hospital) care, or across both (mixed).

Care level	Person inclusion			Values, n (%)
	Disease	Encounter	Population	
Mixed, n	40	34	15	89 (42.4)
Primary, n	2	14	7	23 (11)
Secondary, n	27	69	2	98 (46.7)
Total, n (%)	69 (32.9)	117 (55.7)	24 (11.4)	210 (100)

**Figure 3.**
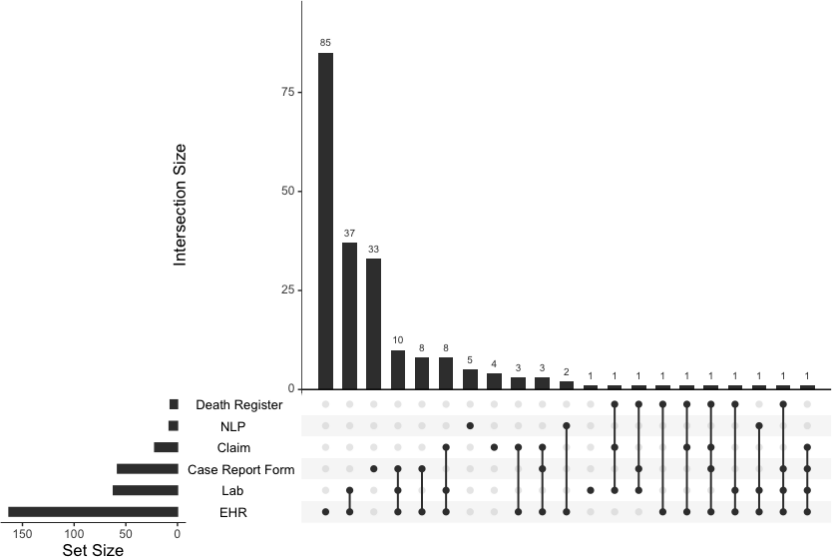
The frequency and overlap of different data capture methods used across 210 standardized real-world data sources in the European Health Data & Evidence Network as of September 1, 2024. EHR: electronic health record; NLP: natural language processing.

Figure 3 shows the number of data sources that receive information through each capture method and each combination of capture methods. EHR is the most common, with 74.7% (157/210) of data sources reporting at least 1 capture method as EHR. Over half of those data sources (85/210) report EHR as their only method for receiving data. Laboratory is the second most common way data sources capture information, as it is reported in 29.5% (62/210) of data sources. Unlike EHR, laboratory data is more likely to be coupled with another data capture method, as only one data source lists laboratory as the singular way they receive information.

## Discussion

### Principal Findings

The varied health care data across Europe, as demonstrated by the summary of 210 data sources in EHDEN from 30 countries, underscores the critical need to generate evidence from more than one data source to comprehensively represent the health care needs or experiences of the entire European population. Across the person inclusion and care levels represented in the network, the data sources are well distributed, emphasizing how health care systems, populations, and data capture methods can differ substantially. While 74.7% (157/210) of the data sources report EHR as at least one of their data capture methods, only 40.4% (85/210) report EHR as their only data capture method. The other 34.3% (72/210) report some combination of EHR, laboratory, case report form, claim, natural language processing, and death register data, showcasing the tremendous heterogeneity of data available in Europe.

### Prior Initiatives

Prior initiatives like European Union–Adverse Drug Reactions (EU-ADR) and Innovative Medicines Initiative–European Medical Information Framework (IMI-EMIF) laid the groundwork for EHDEN, with learnings from those projects directly impacting this project [15,18,36,37]. EU-ADR demonstrated the feasibility of building a federated data network for large-scale drug safety monitoring in Europe using common data analysis files. IMI-EMIF made the first transition from using common input files like those in EU-ADR to the OMOP CDM, but it was not scalable due to the lack of funds and need for trained SMEs, both problems which EHDEN addressed.

### Sustainability and Success of EHDEN

The sustainability of the EHDEN initiative has been achieved through a combination of mechanisms that foster shared leadership, collaboration, and long-term value creation. One key factor has been the stimulation and enablement of both national and European collaborations. The establishment of OHDSI National Nodes has provided a platform for DPs within individual countries to collaborate, share best practices, and enhance data quality [38]. These nodes facilitate national-level harmonization while ensuring compliance with local regulations and coding systems, thereby strengthening the network’s integrity. Beyond this, EHDEN’s adoption in multiple European projects has further expanded its influence, including its pivotal role in enabling large-scale initiatives such as the Data Analysis and Real World Interrogation Network (DARWIN EU). This has also influenced how the European Federation of Pharmaceutical Industries and Associations (EFPIA) is standardizing its data, demonstrating EHDEN’s impact across sectors.

EHDEN has also delivered economic value by creating local ecosystems that support SMEs and DPs. Through the Harmonization Fund, EHDEN has injected resources into the European health care data landscape, with the return on investment yielding a multiplier effect. By recruiting and training SMEs through the EHDEN Academy, the initiative has built local expertise to support DPs throughout the ETL process, ensuring decentralized and sustainable support for the network.

One of the goals of EHDEN has been to standardize health data, akin to utilities like electricity or the internet, essential and accessible to a rapidly growing number of stakeholders across Europe. Now that EHDEN has transitioned from a project under IHI to the nonprofit EHDEN Foundation, the focus has shifted to sustaining, expanding, and improving the network while leveraging the harmonized data for evidence generation. This next phase aims to generate meaningful RWE for research and regulatory purposes. A recent report by The European Commission on the future of European competitiveness highlights EHDEN’s foundational role in shaping the future of the European Health Data Space, further solidifying its legacy as a critical driver of innovation and collaboration in European health care [39].

The success of EHDEN in harmonizing data to the OMOP CDM has led to significant advances in methods research and evidence generation [40-43]. Many of the DPs involved in EHDEN have used their standardized data to conduct analyses across a broad spectrum of use cases. For instance, several studies have been conducted to describe the natural history of diseases, the safety and effectiveness of treatments, and health care utilization patterns across diverse populations [44-46]. One clear example is a multinational network cohort study by Li et al [45], which used evidence generated from EHDEN DPs to characterize background incidence rates of adverse events of special interest related to COVID-19 vaccines. EHDEN’s standardized data has also enabled and improved the development of predictive models, allowing for personalized predictions of treatment outcomes and disease progression [47,48]. The harmonization of data has facilitated large-scale population-level studies, which are crucial for understanding trends in public health and informing health care policy decisions [49,50]. These examples of evidence generation illustrate the broad applicability of the data in the network, which serves both academic researchers and regulatory agencies. A regularly updated list of EHDEN-supported studies and publications is maintained on the EHDEN website, providing a comprehensive overview of the diverse applications of the network in regulatory, clinical, and methodological research.

A notable demonstration of EHDEN’s success is the network’s use in providing timely information on medicines under surveillance due to shortages in multiple European countries. More than 50 DPs contributed data to a study titled “Incidence, Prevalence, and Characterization of Medicines with Suggested Drug Shortages in Europe” [51]. This study represents the largest observational database study conducted across Europe, both in terms of the number of databases involved and its geographic scope. The findings will support European efforts to monitor the use of critical medicines, contributing to the global fight against medicine shortages.

The EHDEN data network has also affected other European collaboratives around RWD. IMI projects like PIONEER, BigData@Heart, EU-PEARL, and HARMONY also use the OMOP CDM and have partly continued the mapping work done in EHDEN [52-55]. In the EMA-commissioned DARWIN EU initiative, among the 20 DPs onboarded in the first 2 years, 16 are also EHDEN DPs [56].

### Future Directions

Building on the progress achieved through the EHDEN network, several key areas offer opportunities for future development. One priority is fostering sustained engagement with DPs. Continuous collaboration will be essential to ensure that DPs remain active contributors to the network by regularly updating and improving their data contributions. Strategies to incentivize engagement, provide ongoing support, and ensure mutual value will be vital for the network’s long-term success, particularly as efforts shift toward more robust evidence generation and ongoing enhancements in data quality.

Expanding the network’s reach and optimizing its databases for specific research use cases are also key areas for growth. With 210 data sources currently included, there is a significant opportunity to onboard additional DPs and expand the network’s coverage across Europe. Future studies will also help identify gaps where further data optimization is required, such as refining mappings or addressing specific quality issues to ensure that the evidence generated is robust, reproducible, and generalizable.

Finally, the newly established EHDEN Foundation will play a critical role in these efforts. By securing funding and fostering collaborations, the Foundation can drive the onboarding of new DPs, address emerging research questions, and ensure that EHDEN continues to adapt to the evolving health care landscape. It also serves as a point of entry for external researchers, who may engage with the network and propose studies through its federated framework. These directions will position the network to remain a cornerstone for RWE generation in Europe, supporting both research and regulatory innovation.

### Conclusions

The results of this study demonstrate that the identification, harmonization, and standardization of data sources through EHDEN have contributed significantly to understanding the diverse RWD landscape and advancement of evidence generation across Europe. These efforts are not only improving observational health research but are also influencing broader regulatory initiatives, such as DARWIN EU, which builds on the foundational work of EHDEN to leverage RWD for regulatory decision-making. Now that the initiative has transitioned to the EHDEN Foundation, there is an opportunity to focus even more on generating high-quality evidence, further solidifying the role of real-world data in improving health care and informing policy decisions across Europe.

## Supplementary material

10.2196/74119 Multimedia Appendix 1 European Health Data & Evidence Network data partner call description.

10.2196/74119 Multimedia Appendix 2 European Health Data & Evidence Network framework for quality benchmarking.

10.2196/74119 Multimedia Appendix 3 European Health Data & Evidence Network subgrant agreement model.

## Abbreviations

DARWIN EU: Data Analysis and Real World Interrogation Network
DP: data partner
DQD: data quality dashboard
EFPIA: European Federation of Pharmaceutical Industries and Associations Declarations
EHDEN: European Health Data & Evidence Network
EHR: electronic health record
EMIF: European Medical Information Framework
ETL: extract-transform-load
EU-ADR: European Union–Adverse Drug Reactions
IMI: Innovative Medicines Initiative
LOINC: Logical Observation Identifiers Names and Codes
OHDSI: Observational Health Data Sciences and Informatics
OMOP CDM: Observational Medical Outcomes Partnership Common Data Model
RWD: real-world data
RWE: real-world evidence
RxNorm: Prescription Normalized Names
SME: small-to-medium enterprise
SNOMED CT: Systematized Nomenclature of Medicine – Clinical Terms
