# Supplementary material for: Advancing Real-World Evidence Through a Federated Health Data Network (EHDEN): Descriptive Study
Source: J Med Internet Res. 2025 Aug 7;27:e74119. doi: 10.2196/74119 (PMC12331365; doi:10.2196/74119)
Supplement: Multimedia Appendix 1 [file jmir-v27-e74119-s001.pdf]

Data Partner

Call Description

The EHDEN Consortium

26/09/2022

V8.1

This project has received funding from the Innovative Medicines Initiative 2 Joint Undertaking (JU) under grant agreement No 806968. The JU receives support from the European Union's Horizon 2020 research and innovation programme and EFPIA.

Table of Contents

1 Document History **2**

2 Document Changes **2**

3 Introduction **3**

3.1 The EHDEN Project **3**

3.2 Why become a Data Partner? **4**

4 Data Partner Calls **6**

4.1 Application Process **9**

4.1.1 Technical Self-Assessment 9

5 Evaluation Process **10**

5.1 Eligibility Check **10**

5.1.1 Admissibility criteria 10

5.1.2 Eligibility criteria 10

5.2 Evaluation **11**

5.2.1 The Data Source Prioritisation Committee 11

5.2.2 Evaluation Criteria 11

5.2.3 Complexity Assessment 13

5.2.4 Independent evaluation 13

5.2.5 Consensus Meeting 13

5.3 Financial Support **13**

5.4 Feedback on the evaluation process **14**

5.5 Appeal Procedure **14**

6 Execution Phase **15**

6.1 Signature of sub-Grant Agreement **15**

6.2 Milestone Payments **15**

6.3 SME Selection **16**

6.4 Data Partner Onboarding **16**

7 Questions **17**

Appendix 1. Application Form **18**

Appendix 2. Eligible Countries **23**

1 Document History

Version	Date	Description
V0.1	21-5-2019	First Draft
V0.2	30-6-2019	ExCom Review
V1.0	10-7-2019	Final Draft Version for public review
V2.0	22-8-2019	Final version for IMI review
V3.0	28-8-2019	Final version
V4.0	31-08-2020	Revised version for 3rd Data Partner Call
V5.0	19-02-2021	Revised version for 4th Data Partner Call
V6.0	16-09-2021	Revised version for 5th Data Partner Call
V7.0	XX-04-2022	Revised version for 6th Data Partner Call
V8.0	19-09-2022	Revised version for 7th Data Partner Call

2 Document Changes

Version	Date	Section	Description
V2.0	22-8-2019	All	References to public review phase removed.
		4	Prescribed budget for the pilot call is set to 2 million Euro. Clarification added on the alternatives to the technical assessment.
		5	Clarification added on the maximum amount of financial support and timelines.
		6	Details added on the collaboration with the SME.
		7	Reference added to the FAQ section on the EHDEN website.
V3.0	30-8-2019	3	Call procedure moved to section 4.
		5	Prioritisation made part of the evaluation step.
		Appendix	Added application form
V4.0	31-08-2020	All	Updated to reflect the 3rd data partner call requirements.
V5.0	19-02-2021	All	Updated to reflect conditions for 4th data partner call.
V6.0	16-09-2021	3,4 and 5	Updated to reflect conditions for 5th data partner call
V7.0	xx-04-2022	Appendix 1	Updated form
		3,4,5	Updated to reflect conditions for 6th data partner call
V8.0	19-09-2022	All	Updated to reflect conditions for 7th data partner call
V8.1	26-09-2022	All	Additional edits for HDR UK call partnership

3 INTRODUCTION

This document describes the Data Partner Call in the European Health Data and Evidence Network (EHDEN) project (www.ehden.eu) that opens at 12:00 CET 12th October and closes 11th November 17:00 CET, in collaboration with Health Data Research UK (HDR UK, www.hdruk.ac.uk) as a funding partner.

In this introduction section we provide information about the EHDEN project, HDR UK, introduce the Data Partner Call, and the value proposition for the Data Partners.

For this final Data Partner call of the IMI phase of EHDEN, HDR UK will also partner to support UK Data Partner applicants specifically.

3.1 The EHDEN Project

The EHDEN project is a public-private partnership set up under the framework of the IMI2 programme (www.imi.europa.eu). The EHDEN consortium consists of twenty-three partners, including academia, Small and Medium-sized Enterprises (SMEs), patient associations, regulatory authorities and pharmaceutical companies. EHDEN is led by the Erasmus Medical Centre, The Netherlands, and Janssen Pharmaceutica N.V., Belgium. The mission of EHDEN is to provide a new paradigm for the discovery and analysis of health data in Europe, by building a large-scale, federated network of data partners across Europe. Central to EHDEN will be the standardisation of health data to the Observational Medical Outcomes Partnership (OMOP) common data model (CDM), and the utilisation of analytical tools such as those developed by the international Observational Health Data Sciences and Informatics (OHDSI) open science collaboration (www.ohdsi.org), and others. The ultimate aim is to facilitate the generation of valid real-world evidence to improve patient care and to enable medical outcomes-based research at an unprecedented scale. The EHDEN consortium will provide the infrastructure and ecosystem to make this ambition come true, supporting academia, healthcare professionals and authorities, patients, pharmaceutical and life sciences companies, and regulatory authorities.

The ambitions of the EHDEN project are high. We aimed to standardise more than 100 million patient records across Europe from different geographic areas and different data sources, e.g., hospital data, registries, population databases, etc., and are now working with >650 million. Standardising such data to the OMOP CDM will facilitate their use for a variety of purposes, enhancing and accelerating research and healthcare decision-making for global benefit.

Through EHDEN, data custodians of longitudinal, person-level observational health data can benefit from financial support to standardise their data to the OMOP CDM and locally install the analytical tools. Once the Data Source is standardised and the tools are operational, the Data Partner becomes a member of the EHDEN community and Federated Data Network and can participate in research studies following their own governance rules.

3.2 Health Data Research UK

Established in April 2018, HDR UK is the UK's national institute for health data science (www.hdruk.ac.uk). Its mission is to unite the UK's health-relevant data to enable discoveries that improve people's lives. HDR UK is an independent charity supported by 10 funders. HDR UK works in partnership with many different organisations across integrated areas of activity covering Research Data Infrastructure and Services and Research Driver Programmes. It convenes the UK Health Data

Research Alliance (https://ukhealthdata.org/) that currently includes over 70 custodians and curators of data, including a network of health data research hubs. HDR UK is also responsible for the development and delivery of the Health Data Research Innovation Gateway (https://healthdatagateway.org/) which provides metadata on over 780 datasets to help data discovery. The gateway also has a nascent cohort discovery functionality that has been developed as part of the COVID - Curated and open analysis and research platform (CO-CONNECT) project. CO-CONNECT requires its data partners to convert their data to the Observational Medical Outcomes Partnership (OMOP) format.

In November 2021, the Data Officers Group of the UK Health Data Research Alliance published a set of recommendations for Data Standards. One of these recommendations was: *"For those custodians who have not adopted a specific data standard/model, HDR UK broadly recommends consideration of the OMOP data model and HL7 FHIRv4 messaging specification standard."*

To support the implementation of this recommendation and build on the progress made through both CO-CONNECT and implementations by the existing 17 UK based EHDEN Data Partners, HDR UK is delighted to be partnering with EHDEN for this Data Partner Call.

3.3 Why become a Data Partner?

Below we list key reasons why we believe a data custodian may wish to become an EHDEN Data Partner:

- **Easier scientific collaboration through a thriving community**
  Having your data mapped to a common data model (CDM) and becoming an EHDEN Data Partner facilitates connections and collaboration with hundreds of peers and exchange of learnings on an unprecedented scale. Methods can be seamlessly shared, and results can more easily be integrated across sources, regions and countries securely. Becoming a Data Partner in EHDEN also means that you can contribute and benefit from an ever-expanding network of researchers and data scientists from all kinds of institutions, including key stakeholders such as HTA and regulatory agencies. A vibrant academic and medical research community awaits you.

- **Boost opportunities to participate in international studies for larger impact**
  The much larger conclusive power of the evidence that can be generated through the EHDEN eco-system will be attractive for everyone. This will mean that your opportunities to participate in large international networks and studies, that may appear as otherwise not accessible now, will be multiplied, as will the impact of any research results derived from such studies. You will also be able to initiate studies yourself so that your own research is amplified and benefits from many other OMOP-mapped datasets of peer Data Partners interested in the same questions.

- **Improved interoperability adds value to your data**
  Common data models such as OMOP are all about interoperability – the capability of systems to speak a common language and understand each other. Mapping to OMOP allows datasets to contribute to generating evidence on a much larger scale. By participating in EHDEN, you expand the value of your data by enabling its re-use across a wide range of analytic use cases, including clinical characterization for disease natural history and quality improvement, population-level effect estimation for safety surveillance and comparative effectiveness, or patient-level prediction for disease interception and precision medicine.

- **Increased visibility for your capacity**

Belonging to the EHDEN eco-system and having your datasets mapped to OMOP CDM will expose your potential to a world of opportunities. You could be approached for sponsored academic studies, trial recruitment, regulatory studies, collaborative European projects, … Whether you are very active already in collaborative initiatives, or just starting to be known, your research capacity will be promoted, potential boosted and network multiplied. These opportunities may help realise the value of your data and secure the financial sustainability of your data source with stable revenue streams.

- **Enhanced capability for study design and analysis thanks to a host of open-source tools**
  Once your data is mapped to the OMOP CDM, you get access to many open-source tools developed by the OHDSI community. These tools will allow you to quickly and consistently design and perform studies, visualise and analyse data. These tools are free and easy to use, and the OHDSI and EHDEN communities include educational resources to learn how to use them and maximise their usefulness in a variety of settings. An EHDEN Academy has been set up to gather and organise educational resources underpinning the eco-system, which will make things even easier.

- **Increased reproducibility and transparency of analyses**
  One of the many advantages of mapping to the OMOP CDM is the fact that methods and analyses can be more easily applied to many data sets for a much larger impact. This increases transparency towards the research and open science community and facilitates reproducibility of studies across diverse settings, increasing the opportunities for rapid learning across the EHDEN eco-system, and boosting the value and confidence in the evidence it generates.

- **Faster performing studies for accelerated research**
  Good research takes time. But the world moves fast nowadays, and human and financial resources are scarce. Efficiency is critical for the survival of any organisation, and this must be achieved without sacrificing quality. Having your data mapped to OMOP CDM means that some analyses can take days instead of months or years, and still use all best practice to generate reliable evidence. This means that you can undertake more studies in less time, decide quicker on whether a research question is futile, explore sensitivity analyses much more efficiently and, in conclusion, be able to generate evidence at a much higher rate – so that any new insight can reach the patient as soon as possible.

- **Federated approach allows to retain full control of the data**
  By supporting a federated approach to the undertaking of studies, EHDEN ensures that data custodians retain control of their data at all times. Scientific independence will not in any way be compromised, nor will your local governance rules, regulations and approval procedures. Your freedom is completely preserved: you still decide in which studies you participate, and you can participate in other networks or projects at your will – no exclusivity is required. You can maintain your current academic and/or commercial interests. The EHDEN eco-system just offers you a lot of additional opportunities – but the final call is always yours.

- **Support your own decision-making processes by easily characterising and visualising your data**
  You know your data. But experience shows that the process of mapping them to a common data model and being able to use them at large can have enormous benefits within your own organisation alone. You will be able to use the OHDSI and EHDEN tools on your dataset, visualise your data in many ways, characterise them, refine coding practices, propagate learnings in your organisation and downstream. In healthcare systems, where strategic decision making heavily depends on reliable data, it may be invaluable to gain real-time insight more rapidly and

inexpensively. If you have multiple datasets, you will be able to manage aggregated views, and analyse them in several ways. You will simply get more out of your own data for your own needs.

- **Platform for training of young researchers and new staff**
  Being part of an active community means that new staff and early career researchers will have numerous ways to learn, quickly and effectively, the skills needed to work with both data mapped to OMOP CDM and open-source tools. This in fact creates a rapid learning environment that alleviates the cost of personnel turnover and makes it easier to preserve the knowledge acquired through the undertaking of studies – so that such knowledge can be consulted and re-used in the future.

- **Framework to demonstrate reliability and utility of observational data analyses**
  For anyone whose research or professional interests are related with the use of observational data, it is important to demonstrate the value of such data, especially in the context of practical real-life scenarios. The eco-system that EHDEN is building aims to help prove the reliability and utility of observational data analyses to generate real-world evidence and, the more proven this is, the more value observational data will have for all kinds of stakeholders.

- **Improve your readiness for a new research environment and evidence generation framework**
  The advent of new environments where rapid evidence generation at scale is required for decision making, be it in healthcare, regulatory, drug development or even in academic settings, is not the distant future. It is here already. Preparing your data for such a connected new world will be the safest way to make sure that data, and the knowledge that it contains, continues to be generated, collected and used for the global benefit of citizens worldwide. Help your data create evidence. Let your evidence help the world.

We highly recommend watching the EHDEN webinars which include conversations with researchers and data custodians that are already part of this exciting journey (Webinars). Also, the presentations given at the OHDSI Symposia are a valuable resource (www.ohdsi-europe.org and www.ohdsi.org) if you want to become more familiar with the vibrant community. A good example of a network study on characterizing treatment pathways at scale can be found in this link, and recent COVID-19 related studies can be found here. Also, contributions to future EHDEN study-a-thons are encouraged, further information on these can be found here.

Video content of three Data Partners and SMEs exploring their ETL experience can be found here.

A Community Manager was appointed to undergo the day-to-day coordination of the progress of the network and milestone achievement, acting as point of contact between Data Partners and EHDEN team members. In addition, bi-monthly Community Calls are organised in order to present and discuss topics of interest for the whole EHDEN Community.

4 DATA PARTNER CALLS

In EHDEN, a fund of EUR 15 million is available to support the data standardisation efforts across Europe for the duration of the project. To ensure a fair, transparent and efficient use of these funds, open Data Partner calls for awarding financial support are periodically launched and communicated through the EHDEN website, newsletter and social media channels and are promoted via e.g., Horizon 2020 and associated Tender portals. For this specific, final call, HDR UK will partner with EHDEN and

support UK applicants, providing a Fund of up to GBP 300,000. The HDR UK funding is subject to the additional HDR UK funding requirements detailed below (page 8 and Appendix 3).

In parallel, EHDEN is training and certifying Small and Medium-sized Enterprises (SMEs) across Europe that will support Data Partners and other stakeholders in standardising and using the data. Periodic open calls for SMEs were launched to obtain a good geographic spread of SMEs and allow data partners to interact with local SMEs as much as possible. The Catalogue of the EHDEN-certified SMEs can be accessed via this link.

If an EHDEN grant is awarded, the Data Partner can use the funding to pay a certified SME to provide support in the standardisation process and installation of the analytical infrastructure or can use this to perform the work themselves if they have the necessary competences. The EHDEN academy is freely accessible by everyone and contains many relevant online courses. Both the data standardisation and analytical infrastructure do however need to at least pass an inspection by a certified SME before the last part of the financial support will be released. The definitive selection of the SME by the Data Partner is only required **after** the financial support has been awarded as shown in Figure 1. Applicants to the Data Partner call **do not** necessarily have to contact any SME at the application stage, nor have to apply jointly with any SME to be awarded the grant. However, they are free to initiate contacts with SMEs at any point in time.

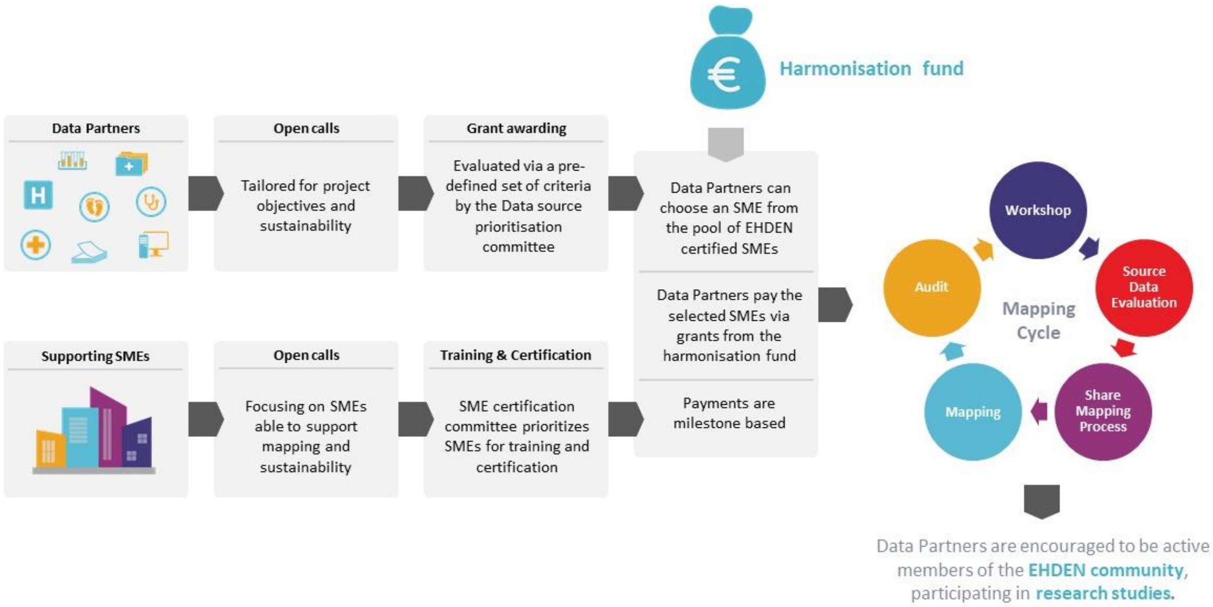

Figure 1. EHDEN's Open Calls for Data Partners (top) and Small and Medium-sized Enterprises (bottom), and how both are subsequently interrelated.

In Data Partner Calls, funding can be requested for the following types of activities:

A. Create new Data Transformation and Analytical Infrastructure

This activity is intended for Data Partners that have not started to map their data to the OMOP-CDM yet. It includes data source profiling, development and implementation of the ETL process and quality control assessments. Additionally, the analytical tools will be deployed at the local site and the data custodian will receive training. After passing inspection for both the data transformation and analytical infrastructure by a certified SME, data source meta data will be entered into the

EHDEN Database Catalogue to reflect this. The Data Partner can then fully participate in the community and data network.

B. Revise Existing Data Transformation and Analytical Infrastructure

This activity is intended for data sources that are already partially mapped to the CDM, to add additional domains (e.g., measurements, procedures) or add new source vocabulary mappings. The financial support will also be used for quality assessment of the ETL, tool deployment, training and the inclusion in the Database Catalogue after passing inspection for both the data transformation and analytical infrastructure by a certified SME. This activity enables these Data Partners to utilise the expertise in EHDEN to further improve and extend their CDM mapping.

C. Inspect Completed Data Transformation and Analytical Infrastructure

This activity, intended for data sources already fully mapped to the CDM, consists of a standardised procedure developed in EHDEN which needs to be applied by a certified SME to ascertain the quality of the ETL and status of the analytical infrastructure. If the quality of the ETL, including its documentation, is at the level required by EHDEN and the deployed tools are successfully tested at the local site, the data source will be added in the Database Catalogue with the information that this validation has been performed. If the quality of the mapping is below the standard, the Data Partner could fix this, and a second inspection will take place. If substantial changes are needed, the Data Partner could apply for additional funding to remedy this (Activity B).

The Data Partner **can only apply for one of the types of activity above per call** but could potentially apply for funding for multiple types of activities during the course of the EHDEN project. However, **the total amount of funding granted for any one institution, counting all types of grants awarded during the EHDEN project, cannot exceed EUR 100,000**. For UK applicants to the HDR UK fund, only activities A & B are permissible up to a maximum of GBP 85,000.

Example: A Data partner could apply to an EHDEN call and be granted EUR 30,000 for a type C activity, and in a future call apply for a type B activity and be granted EUR 70,000. But a Data Partner that applies to an EHDEN call for a type A activity and is granted EUR 100,000 can no longer apply to future calls, since the maximum funding per Data Partner has already been reached.

The call described in this document is the seventh call for Data Partners to receive financial support from the EHDEN project. **The prescribed budget for this call from EHDEN is EUR 1 million to be distributed across all successful applications and including the three activity types (A, B, C) described above.** **The prescribed budget for this call from HDR UK is up to GBP 0.3 million to be distributed across all successful UK-based applications and including two of the three activity types (A, B) described above.**

All applications will be assessed against the same criteria and depending on the quality and volume of applications, EHDEN may fund UK applicants in addition to HDR UK funding envelope in line with previous calls. Where external funding is available for the OMOP mapping of a data source, the EHDEN financial support will be allocated to the next proposal in the list.

Under this process, successful UK applicants undertaking activity types A & B will receive HDR UK funding in the first instance. Specific requirements for successful UK applicants are listed in Appendix 3.

The selected Data Partners will receive all necessary guidance from EHDEN during the mapping phase to guarantee an efficient and high-quality process.

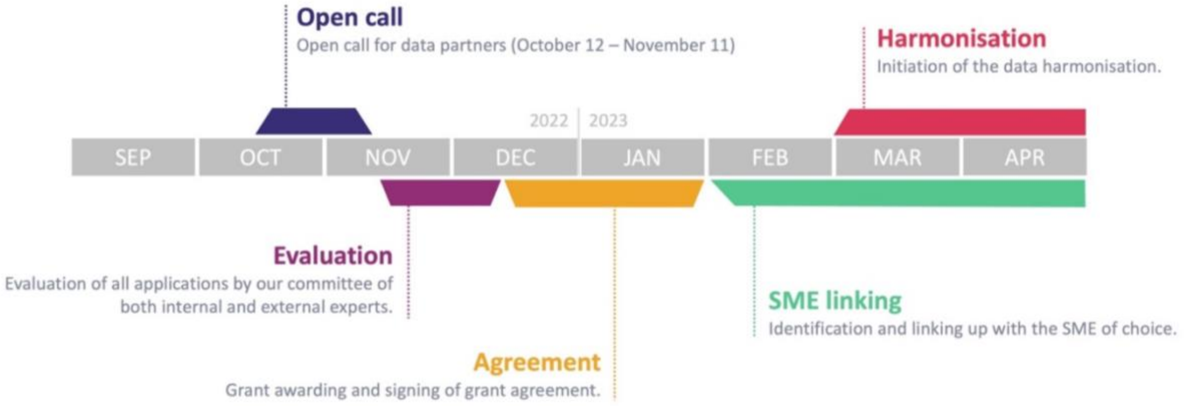

Figure 2. Provisional timelines for the different steps in the call.

The call process includes several steps, described in the next sections. In Figure 2, the (provisional) timelines are presented for the full process: Application, Evaluation, Agreement, SME Linking, and the actual Harmonization process. Formalisation of the grant is made through signature of a Sub-Grant Agreement. EHDEN and HDR UK will both enforce a deadline for sub-grant signature of 6 weeks after the Data Partner is informed of the successful application. The timelines for the harmonization process are agreed upon with the Data Partner in the work plan that is part of the Sub-Grant Agreement, a model of which can be consulted on the EHDEN Website.

4.1 Application Process

The call opens **12th October at 12:00 CET and closes 11th November 17:00 CET**. All applications are to be completed electronically in English through the application portal which will be available on the EHDEN Website, and that will become accessible when the call opens. The application form contains a list of questions that can be found in Appendix 1, so potential applicants are recommended to start preparing their responses in advance of the portal being opened, and in particular run the "Technical Self-Assessment" (see next section) if possible. All applications received before the deadline at the closure date will be reviewed as one batch. Instructions on the use of the application portal can be found on the website.

Applicants should devote sufficient time to properly answer all the sections in the application form. High quality proposals offering a good, complete perspective on the applicant and its potential as data partner invariably fare better during the evaluation process.

4.1.1 Technical Self-Assessment

The application form contains a technical assessment section in which the data custodian is invited to execute the profiling software tool called 'White Rabbit' against the data source. This tool simply extracts information about the data source, the number of tables, the individual fields in the tables, and the frequency of values in the fields. Please review the aggregated data generated by White Rabbit and remove any data you cannot share before uploading in the application portal. This information will

be used to assess the complexity of the data source which will drive the level of maximum financial support (see Section 5.3 below). Furthermore, this self-assessment exercise provides insight in the technical capabilities of the local team to run such a tool and the database readiness for the standardisation exercise. 'White Rabbit' can run locally on the data source so EHDEN does not need any access to the data source. More information about 'White Rabbit' can be found here:

https://www.ohdsi.org/web/wiki/doku.php?id=documentation:software:whiterabbit

If the applicant is not able to run this tool or is unable to share the results, this will not by definition exclude the application from being granted Financial Support – in such a situation, the applicant should however explain in the application form why running White Rabbit or sharing the outcome was not possible. The application form contains in any case other questions that allow evaluators to assess the complexity of the data source.

5 EVALUATION PROCESS

5.1 Eligibility Check

Each application will be first assessed against the admissibility and eligibility criteria described below.

If the proposal is considered inadmissible or ineligible, the applicant will be informed together with the reasons why and how to appeal (see section 5.5).

5.1.1 Admissibility criteria

- The proposal is submitted through the application portal before 11th November, 17:00 CET.
- The proposal is submitted in English.
- The proposal is complete with all relevant fields. Please note that a proposal can be considered inadmissible if most fields are only minimally completed.
- The data partner is legally established and based in an EU Member State or H2020 Associated Country (for a complete list, see Appendix 2). UK applicants to this call are eligible regardless of whether the UK is able to Associate to Horizon Europe.

5.1.2 Eligibility criteria

We welcome all applications, given that:

- The data partner is the legitimate custodian of longitudinal, person-level observational health data of one of the following data source types:
  - Electronic Health Records
  - Claims
  - Hospital
  - Registry
- The data source standardisation should contain Exposures (e.g., drugs, devices), Procedures, and Outcomes (e.g., conditions and measurements).
- Data Sources for which ETLs are already publicly available through the EHDEN or OHDSI platform will not be considered for Activity A, as it is understood that their mapping process is already underway. This is to avoid that multiple Data Partners apply for standardisation of the same Data Source.

Genetic data are currently not captured in the OMOP CDM and will therefore not be considered in the

current round by themselves.

Applications related to other IMI collaborations are appreciated.

5.2 Evaluation

Once the admissibility and eligibility criteria have been checked, the evaluation process will be carried out. A Data Source Prioritisation Committee (DSPC), consisting of experts belonging to the EHDEN Consortium and external institutions, will evaluate each application based on the information available in the application form and the technical self-assessment if provided by the applicant. Applications are assessed on the added value to the network in the context of the applications received. More details on DSPC and the evaluation stages are described in the next sections.

5.2.1 The Data Source Prioritisation Committee

The applications are evaluated by a Data Source Prioritisation Committee (DSPC) which consists at least of 4 EHDEN Consortium members and 4 independent external experts.

The independent experts are drawn from a panel of international experts that act as advisors to EHDEN. All experts involved in the evaluation are required to have the appropriate skills and knowledge to fairly appraise applications. They have a high level of professional experience in the public and/or private sector and are internationally recognised in their field of expertise.

The names of the independent experts assigned to individual proposals are not made public.

An appointment letter binds each independent expert to a code of conduct and establishes the essential provisions regarding confidentiality. The template of this letter and code of conduct can be found on the EHDEN website.

EHDEN takes all necessary steps to avoid conflicts of interest. To this end, all experts are required to sign off for each application that no such conflict of interest (e.g., financial or operational interest in an applicant) exists at the time of their review.

By applying to the EHDEN calls, applicants accept the evaluation and selection procedures established by the project.

5.2.2 Evaluation Criteria

During evaluation, experts are asked to score each application that is assigned to them according to three criteria:

1. **Data impact** (Score 1-10): Score in terms of expected impact of mapping the proposed dataset(s) for the EHDEN eco-system, considering:
   a. Dataset(s) size
   b. Dataset(s) coverage
   c. Perceived quality

   Due to the possibility of multiple applicants with similar or even partially overlapping datasets, the reviewers will be asked to consider the following guiding principles when providing a score:

1. Data sources with more persons within the same data source type get a higher score.
2. Data sources that have more complete coverage from different care settings get a higher score.
3. Data sources that have a more complete representativeness of the underlying population get a higher score.
4. Data sources with more data domains and comparable number of persons get a higher score.
5. Data sources within the same data source type that have longer follow-up time get a higher score.

2. **Network impact** (Score 1-10)**:** Score in terms of expected added value of the Data Partner for the EHDEN eco-system, considering:
   a. Track record of previous studies and collaborations using the data source.
   b. Uniqueness of the type of data versus those already part of EHDEN.
   c. Uniqueness of the regions or countries represented versus those already part of EHDEN.

   All applications are welcome, and from Member States outlined in Appendix 2, applications from countries not yet (or minimally) represented in the EHDEN Network will be especially welcome. Applicants can consult https://www.ehden.eu/datapartners/ for a complete view of the current EHDEN Data Partners, so that they can assess how evaluators may perceive their added value for the Network.

   - Large, national and regional datasets
   - Datasets with granular data will also be especially welcome
   - Datasets with longer term follow up, and in-patient data
   - Including, but not exclusively Patient Outcomes, e.g., PROs/PREMs/HRQoL
   - Including, but not exclusively Claims, resource utilization
   - Including, but not exclusively Registries, small rich datasets

   Specific interest for this call (but not exclusively):
   - All therapeutic areas will be considered

3. **Readiness** (Score 1-10): Score in terms of ability and willingness to actively participate in the EHDEN eco-system, including:
   a. Willingness and ability to participate in federated network studies, including study feasibility assessments.
   b. Availability of information about applicable ethical and governance mechanisms for use of the data in federated network studies.
   c. Willingness and ability to share metadata about the data source to populate the Database Catalogue.

   In relation with metadata, please note that any EHDEN Data Partner will be requested to be included in the Database Catalogue with at least information about the data provenance, available domains of data, database size, univariate statistics on concept level, and the summary findings from the SME inspection. Data sources that are willing to share the ETL documentation will receive a higher score.

Evaluators are asked to clarify and/or justify each of the scores. Their comments must be consistent with any scores awarded and serve as input for any consensus discussion and related consensus report.

The minimum threshold to award an application for each individual criterion is 6. The overall minimum threshold, applying to the sum of the three individual scores, is 20. Applications not reaching these minimum thresholds will not be considered for financial support.

5.2.3 Complexity Assessment

The DSPC will also assess the expected complexity of the data standardisation process based on the information in the application form including the technical self-assessment if provided by the applicant. The committee members are asked to qualify the application in terms of expected complexity of the standardisation effort (Low, Medium, or High). Complexity is mainly used to decide on the grant amount that will be awarded.

If the data source is in a data structure for which an ETL is already available (e.g., the data source has already been mapped by others), the complexity of the mapping is assumed to be lower. If there are source vocabularies that are not yet available in the OMOP Standardised Vocabularies, this will increase the complexity level. The data domains that will be included in the activity will also drive complexity; for example, measurements are much more complex than conditions.

The complexity assessment will include comments to clarify and/or justify the estimated level of complexity. These comments serve as input to the consensus discussion and related consensus report.

5.2.4 Independent evaluation

Each submitted application will be reviewed by at least two randomly assigned DSPC members using the evaluation process outlined above. This includes at least one EHDEN and one independent reviewer per application. Each member will work independently and provide scores and comments according to the evaluation criteria and complexity assessment using the information in the application form.

5.2.5 Consensus Meeting

Once all the DSPC members have completed their individual evaluations, a consensus panel meeting is held. In this meeting, all applications are discussed, and a consensus view is reached on scores, comments and recommendations.

The consensus discussion is moderated by the DSPC Chair. His/her role is to seek consensus between the individual views of independent members without any prejudice for or against a particular application or applicant data source, and to ensure a fair and equitable evaluation of each application according to the defined evaluation criteria.

As a result of this joint revision of applications by the DSPC, all proposals will get a final score and final comments/recommendations. The result of the DSPC assessment will be a list of ranked applications by score for each of the types of activities A, B and C, each application with an expected complexity level (Low, Medium or High).

5.3 Financial Support

Overall, a maximum of EUR 100,000 per Data Partner (or GBP 85,000 for UK applicants funded through HDR UK) can be made available for financial support.
If a Data Partner wants to apply to multiple calls during the EHDEN project, the sum of the financial

fact that one legal entity representing a network will mean the network potentially receiving up to EUR

100.000, which is most probably not constructive. Hence, each legal entity may wish to apply independently within an overall network application. In those cases, it is recommended that reference to the network and connections between applications is made clear by applicants in their respective applications in the portal, but each application will still be considered independently on its own merit.

EHDEN has the public responsibility to make sure it utilises the available funding optimally for the inclusion of as many high-quality data sources as possible without sacrificing equal opportunity and fairness in the process. Therefore, a maximum amount of financial support is set per activity and level of complexity (Low, Medium, High) as shown in Table 1 below. The funding bands are based on prior experience in mapping diverse data sources in other projects, including the OHDSI global collaboration. The main cost components are expected to be the person hours spent on the standardisation process by the applicant and the necessary support services from a certified SME.

Table 1. Financial support per activity

Activity	Max. amount per complexity level in EUR		
	Low	Medium	High
A. Create new Data Transformation and Analytical Infrastructure	40,000	70,000	100,000
B. Revise Existing Data Transformation and Analytical Infrastructure	30,000	50,000	70,000
C. Inspect Completed Data Transformation and Analytical Infrastructure	10,000	20,000	30,000

For UK applicants for HDR UK funding assume equivalent in GBP at rate of EUR 1 to GBP 0.85. Of note, HDR UK funding is not available for Activity C.

EHDEN will assess the price banding of these activities throughout the project based on a fair estimation of actual costs and the feedback obtained from the Data Partners and SMEs. The financial support will be made available to the Data Partners using a milestone-driven approach to obtain maximum control of progress and quality (see section 6 below).

5.4 Feedback on the evaluation process

The EHDEN Project Management Office will send an information email, together with feedback from the Consensus Evaluation relative to the specific application, to all eligible applicants.

The applications found to be ineligible or failing to reach any of the threshold scores for evaluation criteria will be formally rejected by the DSPC. Rejection does not prevent a Data Partner to apply again to subsequent calls.

The applications that reach the threshold scores and are ranked highest in their Activity Category, up to the global funding limit set for this Call, will be deemed successful, will be informed about the amount of financial support offered by EHDEN and be invited to sign a sub-Grant Agreement within specific timelines (see section 6 below). The process for UK applicants who are successful in securing funding from HDR UK be the same as the EHDEN process.

5.5 Appeal Procedure

Applicants have 20 calendar days from the date of sending of the formal decision letter to appeal on the outcome of the evaluation process. This can be done by submitting a reasoned complaint in writing to the EHDEN Project Management Office (PMO) at the email address applicants@ehden.eu. The PMO will confirm receipt and check if all review procedures have been fully respected and no content has been inadvertently omitted or overlooked during evaluation (e.g., due to technical problems with the portal), and will answer to the applicant within 20 calendar days from the date of reception of the letter of complaint. The PMO will not re-evaluate the proposal on the grounds of subjective opinion, and consequently will not call into question the judgement of the appropriately qualified group of experts that form the DSPC.

6 EXECUTION PHASE

6.1 Signature of sub-Grant Agreement

Successful Data Partners will be invited to sign a sub-Grant Agreement with EHDEN that establishes the terms and conditions under which the financial support is offered. The sub-Grant Agreement model is available on the EHDEN Website as part of the documentation of this call and all applicants are strongly encouraged to review it and ensure that the applicant institution will be able to accept the conditions set therein.

A Work Plan in the sub-Grant Agreement will be agreed upon with the Data Partner prior to signature, as well as milestones and an indicative budget. Erasmus MC is the Harmonisation Fund Holder in EHDEN and will sign the agreement on behalf of the EHDEN Consortium. Signature of the sub-Grant Agreement needs to be timely achieved as a pre-requisite to any financial support. To this end, specific deadlines for completion of the tasks leading to sub-Grant Agreement signature will be indicated in the information sent to successful applicants. Failure to adhere to such deadlines may result in withdrawal of the financial support offer by EHDEN.

The process for signature of the Sub-Grant Agreement for UK applicants who are successful in securing funding from HDR UK will be the same as the EHDEN process but in addition Annex 1 of the Work Plan will contain an express statement that the award is funded by HDR UK and subject to the HDR UK Additional Requirements.

6.2 Milestone Payments

The sub-grant agreement will contain a milestone-based schedule for payment of the grant. By default, the following milestone payments for all three (A, B, C) type of activities are proposed:

Milestone	Grant Percentage	Estimated Timelines*
Shared ETL documentation	30%	Month 3
ETL Implemented and Infrastructure Operational	40%	Month 6
Database catalogue entry following final inspection by certified SME	30%	Month 7

*starting at the date of signature.

These milestones are indicative and are understood as the default option. Adjustments in timelines and milestones will be possible and negotiated with each Data Partner on a case-by-case basis prior to signature of the sub-Grant Agreement. The Data Partner will have to submit the milestone artefacts,

e.g., the ETL documentation, and final SME inspection report, through the application portal. Milestones have to be signed off by EHDEN before the corresponding milestone payment is made.

The fulfilment of the agreed timelines as detailed in each workplan if of utmost importance, especially for Call 7, in order to ensure that the mapping can be completed within the EHDEN period. For this reason, all Data Partners are expected to finish their work by the end of February 2024 to guarantee that all payments can be processed.

Data Partners should address all preparatory work before the signature of the Sub-Grant Agreement to avoid any internal obstacle that hinders the execution of the project in the agreed period.

6.3 SME Selection

The standardisation process to the OMOP CDM is a team effort that requires advanced knowledge about the OMOP CDM and should therefore preferably be performed in close collaboration with a certified SME. The Data Partner can use the financial support provided by EHDEN to pay for certified SMEs services, and to cover additional internal costs. If the Data Partner has internal expertise in standardising data to the OMOP CDM, part of the funding can be used by the Data Partner to implement the ETL. The Data Partner can also follow online training through the EHDEN Academy for this purpose. This will be discussed with the Data Partner on a case-by-case basis and will be described in the Work Plan that is part of the sub-Grant Agreement. However, at a minimum, a certified SME is required to perform the final inspection.

EHDEN-certified SMEs have received training from EHDEN on the following topics:

a. Expertise on the OMOP CDM and the Standardised Vocabularies.
b. Knowledge about the Extract, Transform, and Load steps and their implementation with OHDSI tools and approaches developed in EHDEN: http://www.ohdsi.org/data-standardization/
c. Understand the fundamentals of proper documentation of the ETL process in close collaboration with a data custodian, to assure transparency and reproducibility.
d. Expertise in the installation and use of the OHDSI Tools for federated data analyses such as those described here: https://www.ohdsi.org/analytic-tools/

The Data Partner is free to contact and select SMEs from the pool of certified SMEs made public in the SME Catalogue on the EHDEN Website. **EHDEN does not intervene in the selection, contractual arrangements or pricing agreed between Data Partners and SMEs.**

For the avoidance of doubt, a Data Partner DOES NOT have to apply for financial support together with a certified SME. The selection process from the list of certified SMEs can be initiated before or after the financial support is granted. However, procurement processes in some institutions can be lengthy, and therefore applicants are encouraged to start discussions with certified SMEs at their earliest convenience.

6.4 Data Partner Onboarding

After successful completion of the Work Plan, the Data Partner will be part of the EHDEN community and be able to fully utilise all key values as described in the value proposition (section 2.2). Furthermore, we like to highlight the following benefits:

1. **Exclusive invitations for events**

   The Data Partner will receive personal invitations to OHDSI and EHDEN events. This can include invites to symposia or invites to participate in study-a-thons in which researchers and data

sources will collaboratively execute studies to generate reliable evidence. Furthermore, EHDEN will organize data network meetings for the Data Partners to stimulate interaction with other key stakeholders.

2. **Education and training**

   Anyone can access the EHDEN Academy for continuous training, but we aim to offer face-to-face training throughout Europe from leading researchers in the EHDEN and OHDSI community on how to design and execute your own research.

3. **Research study invitations**

   EHDEN Data Partners will be kept up to date on the progress of ongoing network studies and opportunities to participate in new studies. Additionally, EHDEN will provide support when the Data Partner wants to initiate a network study.

4. **Exclusive EHDEN Forum Membership**

   A private section in the EHDEN forum will be accessible to the EHDEN Data Partners to interact directly with other Data Partners and EHDEN Consortium members.

7 QUESTIONS

If you have questions, please send us an email at applicants@ehden.eu. We have also created a Frequently Asked Questions section on the EHDEN Website that you may find helpful.

If you like to stay informed about EHDEN and upcoming calls, please sign up for the newsletter on the website.

www.ehden.eu @IMI_EHDEN IMI_EHDEN github.com/EHDEN

Appendix 1. Application Form

Question	Hints/Options
**Data Source Description**	
Please state the exact name of the legal entity that would sign the grant agreement if your application is successful.	
Provide a brief description of the data source (max 250 words)	Provide the data source introduction you would normally use in a publication.
If this data source is part of an existing network, please describe that network	For example, hospitals that are part of an existing hospital network or registries that already collaborate. This allows us to identify linked applications.
Provide links to max of 5 publications or publicly available links that describe the data source and demonstrate its use.	Please add links to publications whenever possible onto form
From which country is the data originating?	
Describe the geographic coverage.	For example, a certain region or city
Is the data source containing patients with a specific socioeconomic status?	For example, Medicaid is a state and federal program that provides health coverage if you have a very low income.
Is the data source containing patients from a specific health system (insured/uninsured, public vs. private)	We refer here to all the patients in the data source, e.g., a private insurance data source.
What care setting(s) are covered?	• Inpatient (hospital) care<br>• Outpatient general practitioner care<br>• Outpatient specialist care<br>• Long term/ skill nursing facility care<br>• Pharmacy care<br>• Other (free text)
What data capture process(es) are used?	• Insurance/administrative claims<br>• Outpatient electronic health records<br>• Inpatient hospital electronic health records<br>• Inpatient hospital billing systems<br>• Registries<br>• Biobank<br>• Other (free text)
Specify the inclusion criteria for patients to enter the population, if any.	
When did the data collection start?	
Is there any lag in the data capture?	
What is the frequency of source data updates?	
Which of these age categories are included in your data source?	• infants and toddlers (0 days to 23 months)<br>• children (2 to 11 years)<br>• adolescents (12 to 17 years)

	• 18 to 45 years<br>• 46 to 65 years<br>• 66 years and over
What is the gender of the data source population?	• Male<br>• Female<br>• Both
What is the approximate total (cumulative) number of subjects in your data source?	
What is the current approximate number of active patients?	
What is the average follow-up period per patient in years?	
For a description of the data domains in the CDM see the [*CDM Page*]	
Specify all data domains that are covered by your source database.	• Person<br>• Observation Period<br>• Visit Occurrence<br>• Visit Details<br>• Condition Occurrence<br>• Death<br>• Drug Exposure<br>• Procedure Occurrence<br>• Device Exposure<br>• Measurement<br>• Observation<br>• Specimen<br>• Survey<br>• Provider<br>• Location<br>• Care Site<br>• Payer Plan Period<br>• Cost
Does your data source contain vaccines?	• Please add details on what type of vaccines, e.g., COVID-19 vaccines
Does your data source contain COVID-19 cases?	• Please add the approximate number of patients with COVID-19
Does the data source contain free text?	If yes:<br>In what language is the free text?<br>Explain how you are using the free text in your current studies.
Are there any additional comments you like to make about the available data?	
**Data Source Governance and Ethics**	
Is your institute the data custodian?	Please explain if you are the "Owner" of the data source or acquired the data source from another party, or simply have access to it
**Information about the Governance Board procedures for federated data analysis, including timeliness.**	

Do you have an ethical committee / governance board?	
Do you have a publicly available document that details the procedure by which approval can be requested?	
Describe briefly the process of obtaining approval	
What is the average duration of the approval process?	
Which studies have to be registered to ENCePP?	
Are there any areas of use or research that have been explicitly barred by approval bodies or participants?	
Are you allowed to use this data in collaboration with external parties?	For example, mention if this is only allowed with academics
Are you authorized to share aggregated analysis results for research purposes?	
Are you able to contact the health care provider to obtain additional information about the patient?	
Are you able to contact the patient to obtain additional information?	
Are you able to obtain biological samples from the patient?	
Are you able to gather additional information about the patients (for example, environmental data documenting the air pollution in the area where the patient lives)	
**Technical Details**	
What database management system is used for your data source?	If other management system, please specify
Are all your data currently co-located in one database?	
Do you have a document available that describes the database structure?	*Do not upload patient data*<br>If yes, can you share this document. If yes, please upload the document.<br>If no, please explain why not.
Which terminology systems are used in your database?	For example, ICD9, ICD10, local coding system etc.
Are you willing to share the Extraction Transform and Load Document for your data transformation with the EHDEN project?	
If possible, we like to obtain information about the structure of the source database using a profiling tool called White Rabbit. This tool will extract all the table names, their fields, and most frequent values in the fields. Your data needs to be stored in a single relational database management system that is supported by the tool. If you need support send us an email: applicants@ehden.eu	

Note that if you are not able to execute the tool or share its results, this will not by definition exclude your data source from the program. It would however be very useful for the committee to understand the complexity of your data source.	
Are you able to execute the tool?	If not, please explain why
Are you able to share the results of the tool?	If not, please explain why. If yes, please upload.
Explain how you will manage the updates of the CDM in the future?	For example, how frequent will you update? Will you have data managers available to do this? etc.
**Interest and ability to participate in distributed research**	
EHDEN will maintain a public database catalogue that contains contact information and meta data. This meta data consists of information like the governance procedure, but also aggregated data such as total number of patients over time, cumulative patient time etc. We like to know what information you are able to share.	
Do you want to share contact information?	
Please specify what type of aggregated data can be shared once mapped.	
Do you intend to ask research questions and design network studies to generate evidence from the rest of the network? Please explain.	
Do you intend to use the OHDSI tools to conduct research locally?	
Do you plan to participate in network studies lead by others in the network?	
What type(s) of staff at your site are commonly used to conduct database analyses and would likely be involved in federated research?	• DB Admins<br>• data managers<br>• informaticians<br>• statisticians<br>• epidemiologists<br>• clinical researchers<br>• other (free text)
Are you already involved in the OHDSI community? If so, please describe how.	
**Activity selection**	
Select the activity you are applying for.	For more details see the Call Description Document<br>• Create new Data Transformation and Analytical Infrastructure<br>• Revise Existing Data Transformation and Analytical Infrastructure<br>• Inspect Completed Data Transformation and Analytical Infrastructure
For the last two options additional questions are asked:	
Describe which tables have already been populated in your OMOP CDM.	

Describe the status of the source code mapping for the domains you have populated.	
Explain how you like to revise or extend the existing data transformation.	
Do you have documentation of the current data transformation?	If yes, can you share this document. If yes, please upload the document.<br>If no, please explain why not.
Describe the current status of the analytical infrastructure.	Which OHDSI tools and methods libraries are currently used on your data?
Describe who implemented the Extraction, Transform, and Load (ETL) procedure for your current CDM version	For example, a specific SME, internal experts.
**Motivation**	
Describe why you want to apply for financial support and why your data would be of interest to the research community.	
**Signature**	
Are you able and willing to go through the EHDEN application evaluation procedure, described in the call text?	
Declaration	Check box: i am fully authorized to submit this application on behalf of the data partner.
Do you confirm you agree with the Terms of Service and Privacy Policy?	Check box: I agree
Do you agree with the Terms of Service?	Check box: I agree
Can we add you to our newsletter so you will be notified of major milestones of EHDEN and future open calls.	
Can we mention your database name in the project communications (e.g., on the website, newsletter, ...)?	
Signature	Fill in the full name of the applicant

APPENDIX 2. ELIGIBLE COUNTRIES

To be eligible for financial support, the Data Partner should be legally established and based in an EU member state or H2020 associated country for the duration of the sub-grant agreement. Despite uncertainties related to Brexit, the United Kingdom remains an Associate Country for IMI programmes such as EHDEN, and applications are still valid.

EU Member States

Austria	Estonia	Italy	Portugal
Belgium	Finland	Latvia	Romania
Bulgaria	France	Lithuania	Slovakia
Croatia	Germany	Luxembourg	Slovenia
Cyprus	Greece	Malta	Spain
Czechia	Hungary	Netherlands	Sweden
Denmark	Ireland	Poland	

H2020 Associated countries

Albania	Georgia	Montenegro	Switzerland
Armenia	Iceland	North Macedonia	Tunisia
Bosnia and Herzegovina	Israel	Norway	Turkey
Faroe Islands	Moldova	Serbia	Ukraine
United Kingdom			

APPENDIX 3. REQUIREMENTS FOR SUCCESSFUL UK APPLICANTS

Successful UK applicants undertaking activity types A & B (see 5.3) will receive HDR UK funding and will be required:

- to acknowledge and agree that EHDEN may share all information received by it in relation to the UK EHDEN Data Partner and its participation in the EHDEN programme with HDR UK, subject to appropriate confidentiality obligations;
- when promoting the work plan and its major results, to display the HDR UK logo and include text provided by HDR UK and EHDEN to the UK EHDEN Data Partner acknowledging the contribution of HDR UK and its funders to the project;
- to publish results of the research funded by the HDR UK in accordance with normal academic practice and the UKRI policy on Open Access available at https://www.ukri.org/files/legacy/documents/rcukopenaccesspolicy-pdf/.
- to make the HDR UK funded datasets available on a FAIR (Findable, Accessible, Interoperable and Reusable) basis in accordance with applicable legal, ethical, and regulatory requirements and findable through the HDR UK Innovation Gateway; and
- to declare their willingness to support HDR UK by attending its public events and by presenting the results of the Work Plan.

(the "HDR UK Additional Requirements).
