# Supplementary material for: Advancing Real-World Evidence Through a Federated Health Data Network (EHDEN): Descriptive Study
Source: J Med Internet Res. 2025 Aug 7;27:e74119. doi: 10.2196/74119 (PMC12331365; doi:10.2196/74119)
Supplement: Multimedia Appendix 2 [file jmir-v27-e74119-s002.pdf]

EUROPEAN HEALTH DATA & EVIDENCE NETWORK

806968 – EHDEN

European Health Data & Evidence Network

WP4 – WP Technical Framework

D4.6 Final version of the Framework for quality benchmarking

**Lead contributor**	Clair Blacketer (1/12 – EMC/Janssen)
**Lead contributor email**	mblacke@its.jnj.com
**Other contributors**	Peter Rijnbeek (1, EMC)<br>Michel van Speybroeck (12, Janssen)<br>Maxim Moinat (6, The Hyve)

**Due date**	2020-11-30
**Delivery date**	2021-01-11
**Deliverable type**	R
**Dissemination level**	PU
**DoA - Version**	V1
**Date**	2018-12-11

This project has received funding from the Innovative Medicines Initiative 2 Joint Undertaking (JU) under grant agreement No 806968. The JU receives support from the European Union's Horizon 2020 research and innovation programme and EFPIA.

TABLE OF CONTENTS

Table of contents 2

Document History 3

Definitions 4

Publishable Summary 5

1. Introduction 6

2. Data Quality Dashboard 6

2.1 Motivation 6

2.2 Development 7

2.2.2 DQ Checks 7

2.3 Dashboard usage 7

2.4 Implementation 8

2.5 Year Two Updates 9

2.5.1 DQD on a Cohort 9

2.5.2 Notation on Data Quality Checks 9

2.5.3 User-Interface Changes 9

3. Next Steps 9

4. Conclusion 10

© Copyright 2020 EHDEN Consortium

Document History

Version	Date	Description
V1	09 Nov 2020	First Draft for review
V2	08 Dec 2020	Second draft for formal review
V3	10 Dec 2020	Third draft for consortium review
V3final	22 Dec 2020	Final format version

© Copyright 2020 EHDEN Consortium

DEFINITIONS

Participants of the EHDEN Consortium are referred to herein according to the following codes:

**EMC**	Erasmus Universitair Medisch Centrum Rotterdam- The Netherlands (Project Coordinator)
**Synapse**	Synapse Research Management Partners S.L. - Spain
**UOXF**	The Chancellor, Masters and Scholars of the University of Oxford - United Kingdom
**UTARTU**	Tartu Ulikool - Estonia
**UAVR**	Universidade de Aveiro – Portugal
**The Hyve**	The Hyve BV – the Netherlands
**Odysseus**	Odysseus Data Services SRO – Czech Republique
**EPF**	Forum Europeen des Patients (FPE) - Belgium
**NICE**	National Institute for Health and Care Excellence – United Kingdom
**UMC**	Stiftelsen WHO Collaborating Centre for International Drug Monitoring - Sweden
**ICHOM**	International Consortium for Health Outcomes measurement LTD - United Kingdom
**Janssen**	Janssen Pharmaceutica NV - Belgium (Project Lead)
**Pfizer**	Pfizer Limited – United Kingdom
**Abbvie**	AbbVie Inc - United States
**IRIS**	Institut De Recherches Internationales Servier - France
**SARD**	Sanofi Aventis Recherche & Developpement - France
**Bayer**	Bayer Aktiengesellschaft - Germany
**Lilly**	Eli Lilly and Company Limited – United Kingdom
**AZ**	AstraZeneca AB - Sweden
**Novartis**	Novartis Pharma AG - Switzerland
**UCB**	UCB Biopharma SPRL - Belgium
**Celgene**	Celgene Management SARL - Switzerland

**Grant agreement**	The agreement signed between the beneficiaries and the IMI JU for the undertaking of the EHDEN project (806968).
**Project**	The sum of all activities carried out in the framework of the Grant Agreement.
**Consortium**	The EHDEN Consortium, comprising the above-mentioned legal entities.
**Consortium agreement**	Agreement concluded amongst EHDEN participants for the implementation of the Grant Agreement. Such an agreement shall not affect the parties' obligations to the Community and/or to one another arising from the Grant Agreement.

© Copyright 2020 EHDEN Consortium

PUBLISHABLE SUMMARY

The Data Quality Dashboard (DQD) developed as part of EHDEN Work Package 4 provides a comprehensive, customizable, and transparent way to both evaluate and communicate the quality of an OMOP CDM instance. It provides the code to run data quality checks against an OMOP CDM instance, and a way to visualize the results in a web application. The DQD is described in deliverable 4.2; this deliverable provides an update on progress and details of what was achieved in year two.

Each Data Partner runs the Data Quality Dashboard on the data at their site once it is converted to the OMOP CDM. Initially, it has been used to assess whether OMOP Standards such as primary key constraints and concept domain restrictions are being followed. Thinking specifically of the EHDEN federated network, providing an interactive data quality report for each participating site provides evidence not only that OMOP specifications were followed correctly but also demonstrates that the necessary due diligence was performed to ensure that the data are of research quality.

As the EHDEN network continues to grow and more Data Partners are at the point where they can run the DQD tool on their data we have been able to use it to assess adherence to CDM standards and readiness for network research. Such practical application will lead to continued innovation as we have already seen during year two. During the recent Rapid Collaboration Call for COVID-19 data sources, the DQD has been invaluable, providing insight into issues during the extract, transform, and load process and even serving as an education tool to disseminate standards and expectations.

The DQD has already been widely adopted across multiple observational health networks in addition to EHDEN. The National COVID Cohort Collaborative[1], funded by the US National Institutes of Health, is building a comprehensive database of COVID patients from across the US. They employ the tool during their data ingestion and harmonization process to assess data as it is received. The US FDA Biologics Effectiveness and Safety System[2] uses the DQD to evaluate data quality prior to engaging in observational research. Similarly, the OHDSI community has begun to require Data Partners to run the tool prior to participation in network research. In response to this support and usage a publication is in production to fully describe the tool and to increase dissemination. The DQD has proven to be a strong foundation for data quality reporting and a key tool to ensure confidence in the evidence generated through the EHDEN federated network and beyond.

[1] https://ncats.nih.gov/n3c

[2] https://www.fda.gov/vaccines-blood-biologics/safety-availability-biologics/cber-biologics-effectiveness-and-safety-best-system

© Copyright 2020 EHDEN Consortium

1. Introduction

This document reports on the development of a systematic approach to quality assessment of data converted to the OMOP CDM, delivered by the European Health Data and Evidence Framework (EHDEN).

The definition of data quality is 'a measure that validates the actual behaviour of a "component" against its original intended characteristics of use in operations, decision-making, and planning'[1]. This means that a data source can be of high quality for one purpose and of low quality for another. In addition, the same data quality metric can mean different things for different data sources, e.g., missing race information is inherent in some data sources as this is not always captured.

The conversion of source data to the OMOP CDM (extract-transform-load, ETL) is a multi-step process which requires a team with diverse skills and knowledge. Data quality checking is required at every step of the process. During the mapping implementation step, this can be achieved with unit tests to check the logic employed to convert the source data structure to the CDM. After the transformation step has been completed, the data quality can be checked by running an assessment. In previous years the OHDSI community used a report called 'Achilles Heel', a component of a tool called ACHILLES that both characterizes the data in the OMOP CDM and runs a set of data quality checks. However, the number of data quality checks were limited, not customizable and it was difficult to find the cause of a check failure.

In the context of EHDEN and the wider OHDSI community, the Data Quality Dashboard was developed in response to this need and has been in use for over a year. This dashboard includes over 3,300 automated quality metrics and gives detailed information on failed data quality metrics. The output can be tailored for each data source to make sure that relevant metrics are captured. The tool itself is widely adaptable and can be expanded easily to include any number of additional quality checks. Over the last year alone multiple features have been added, on request of EHDEN, to make it easier to use and more applicable to research.

The DQD has already been widely adopted across multiple observational health networks in addition to EHDEN. The National COVID Cohort Collaborative[3], funded by the US National Institutes of Health, is building a comprehensive database of COVID patients from across the US. They employ the tool during their data ingestion and harmonization process to assess data as it is received. The US FDA Biologics Effectiveness and Safety System[4] uses the DQD to evaluate data quality prior to engaging in observational research. Similarly, the OHDSI community has begun to require Data Partners to run the tool prior to participation in network research. In response to this support and usage a publication is in production to fully describe the tool and to increase dissemination.

2. Data Quality Dashboard

2.1 Motivation

The goal of the Data Quality Dashboard (DQD) project was to design and develop an open-source tool to evaluate observational data quality in response to the needs of global regulatory agencies.

[3] https://ncats.nih.gov/n3c

[4] https://www.fda.gov/vaccines-blood-biologics/safety-availability-biologics/cber-biologics-effectiveness-and-safety-best-system

© Copyright 2020 EHDEN Consortium

2.2 Development

At a high-level, this project consisted of creating a way to run a series of data quality checks against an OMOP CDM instance. The vision was to create an application that would systematically run the checks, evaluate the checks against some pre-specified threshold, and then communicate what was done in a transparent and easily understandable way.

Beginning with the checks themselves, the Kahn Framework[5] was chosen to organize those covered by the tool as it is widely accepted as the terminology to use when discussing data quality. Under this framework, checks fall into categories, subcategories, and contexts.

As described by Kahn et al, there are three categories into which data quality checks can be organized: conformance, completeness and plausibility. Each category can be interpreted within two different contexts that represent strategies for assessing data quality:

- Verification relates to how well data conform to local knowledge, metadata descriptions, and system assumptions.
- Validation relates to how well data align with external benchmarks with expectations derived from known true standards.

2.2.2 Data Quality checks

Using the Kahn framework as the way to organize ideas, the Data Quality Dashboard takes a systematic approach to run data quality checks. Instead of writing thousands of individual checks, we use what we call “data quality check types”. These “check types” are more general, parameterized data quality checks into which OMOP tables, fields, and concepts can be substituted to represent a singular data quality idea.

Version 1 of the tool[6] includes 20 different check types organized into Kahn contexts and categories. Additionally, each data quality check type is considered either a table check, field check, or concept-level check. Table-level checks are those evaluating the table at a high-level without reference to individual fields, or those that span multiple event tables. These include checks making sure required tables are present or that at least some of the people in the PERSON table have records in the event tables. Field-level checks are those related to specific fields in a table. The majority of the check types in version 1 are field-level checks. These include checks evaluating primary key relationship and those investigating if the concepts in a field conform to the specified domain. Concept-level checks are related to individual concepts. These include checks looking for gender-specific concepts in persons of the wrong gender and plausible values for measurement-unit pairs. The full list of check types, the check level (table, field, concept), a description of the check, and Kahn category and context they fit into can be found on the [7]

2.3 Dashboard usage

Within the Data Quality Dashboard package, there is a set of CSV files that dictate how the dashboard should be run and which thresholds should be applied to each data quality check. The ‘*_Check_Descriptions.csv*’ contains the data quality check types, their descriptions, and which SQL file is associated with each. They are

5 https://www.ncbi.nlm.nih.gov/pmc/articles/PMC5051581/

6 https://github.com/ohdsi/dataqualitydashboard

7 https://ohdsi.github.io/DataQualityDashboard/articles/CheckTypeDescriptions.html

© Copyright 2020 EHDEN Consortium

then organized into table-level, field-level, and concept-level checks, each represented with its own CSV file that details which checks are run by placing a ‘Yes’ or ‘No’ in the corresponding cell. Each check has a threshold against which the resulting statistic of the check will be evaluated that can be pre-specified by writing the threshold value in the appropriate cell.

These files have a default structure that is set up to run 3,351 checks using the systematic approach listed in the above section, taking anywhere from a few minutes to a few hours depending on database size. Ideally, all checks would be run on every CDM instance but not all sites can populate every table (natural-language processing data is not always available, for instance). Fortunately, this underlying structure is customizable so that each data partner can adjust which checks are run and how they are evaluated to meet their needs and reflect the aspects unique to their data. This includes turning off checks for CDM tables that are not populated or adjusting the evaluation thresholds. Perhaps only 10% of the records in a MEASUREMENT table have values and the threshold needs to be lowered to reflect that nuance of the data. Each change that is made is then communicated in the final output object.

It is important to note that it is not expected that the default threshold settings will work for every site that uses the DQD. That is why all of the checks that were run are included in the final output and why all pertinent information is captured including the numerator and the denominator.

Once the CSV files are edited based on the SME or data partner’s needs, an R package is called that reads in the CSV files and applies the specifications to run the data quality checks. These results can be written to a table if so desired, but the main output is a JSON object. This JSON object is then used by the Data Quality Dashboard Shiny application viewer to display an interactive set of data quality results. A live demo of this application can be found here.

Figure 1 shows the Overview page of the DQD. It shows a count of all data quality checks, both passed and failed, organized by Kahn category and context. This should be used as a way to understand the potential weaknesses of the database and areas that should be investigated.

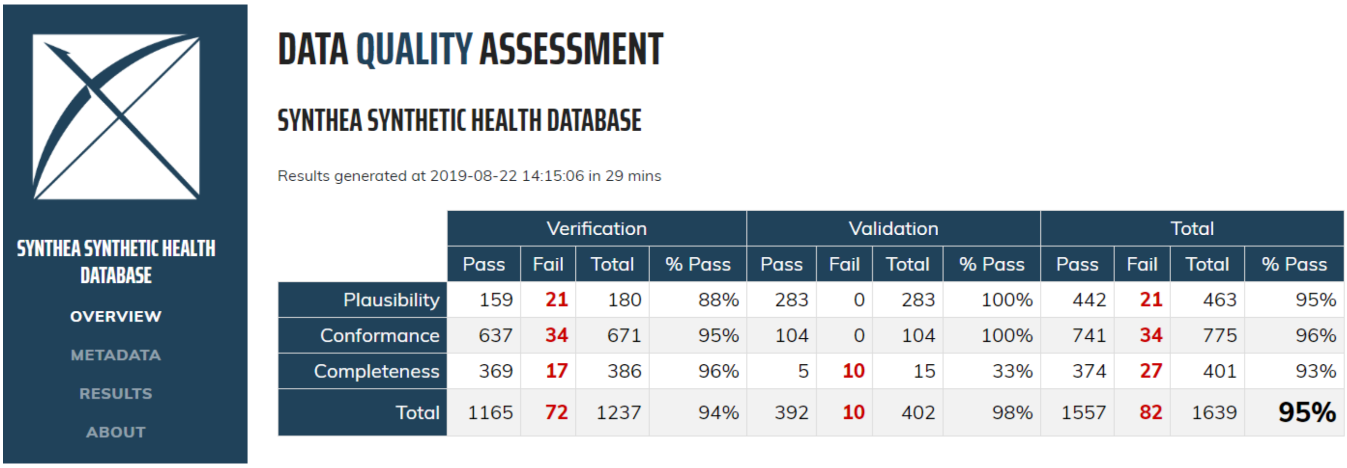

	Verification				Validation				Total			
	Pass	Fail	Total	% Pass	Pass	Fail	Total	% Pass	Pass	Fail	Total	% Pass
Plausibility	159	21	180	88%	283	0	283	100%	442	21	463	95%
Conformance	637	34	671	95%	104	0	104	100%	741	34	775	96%
Completeness	369	17	386	96%	5	10	15	33%	374	27	401	93%
Total	1165	72	1237	94%	392	10	402	98%	1557	82	1639	95%

Figure 1: Data Quality Dashboard Overview Page

2.4 Implementation

Each Data Partner runs the Data Quality Dashboard on the data at their site once it is converted to the OMOP CDM. Initially, it is used to assess whether OMOP Standards such as primary key constraints and concept

© Copyright 2020 EHDEN Consortium

domain restrictions are being followed. It has been invaluable during the EHDEN Rapid Collaboration call to help Data Partners identify issues in their mapping to the OMOP CDM and to help pinpoint where they should spend their efforts. Thinking specifically of the EHDEN federated network, providing an interactive data quality report for each participating site provides evidence not only that ETL processes were performed correctly but also to demonstrate that the necessary due diligence was undertaken to ensure that the data are of research quality.

2.5 Year Two Updates

Over the course of year two, many EHDEN data partners have implemented the DQD and provided valuable feedback that has been incorporated back into the tool. As Data Partners join the federated network they work with a Small to Medium-sized Enterprise (SME) that is certified by EHDEN to convert their data to the OMOP Common Data Model. The DQD is run on the resulting CDM instance and any issues found are addressed and the DQD is run again. This iterative inspection process not only trains the data owners to evaluate their data in a standardized way but the learnings also drive further optimization in the tool itself. The flexibility of the Data Quality Dashboard enables accelerated development and innovation as the tool is constantly evaluated. Version 1 of the tool described in deliverable 4.2 was already very advanced and only slight modifications were suggested, as listed below, which resulted in development of version 1.3 in year two.

2.5.1 DQD on a Cohort

An option has now been added to the Data Quality Dashboard to enable running the tool on a cohort as opposed to the entire database. When approaching data quality from a study perspective, often it is more important to understand the quality of data for a particular cohort than for the whole database.

2.5.2 Notation on Data Quality Checks

After discussions with many data partners during the EHDEN COVID-19 Rapid Collaboration Call they expressed a need to be able to make notations on each data quality check, either to describe why a failure threshold was changed or to explain upcoming changes to the extract, transform, and load process. This feature is now available in the tool's user-interface.

2.5.3 User-Interface Changes

After using the DQD, Data Partners asked for some changes to the user-interface that would make the results easier to read. These updates included adding the table and field names where the data quality check was evaluated as well as the name of the data quality check type.

3. Next Steps

Following the work that was done to develop version 1.3 of the Data Quality Dashboard, a number of items were identified as potential next steps for version 2.

- Add additional vocabulary checks. The DQD can be easily adapted to more tables and fields and it would be valuable to add vocabulary checks that look for potential errors that can arise during download and implementation of the tables.

© Copyright 2020 EHDEN Consortium

- Ease the process for adding data quality checks. The current process for adding checks includes manipulation of the R code. An easier process should be developed so it is merely a matter of adding a new check to the CSV files.
- Develop a way to visualize data quality results across a network. The Shiny application currently only shows the DQD results from one database. It would be helpful to see the results of a network of databases so that they can be compared.
- Create a way to edit the check thresholds without restarting the entire process. Currently, if failure thresholds are changed, a full rerun of the tool is necessary for them to take effect. Multiple Data Partners have requested a change to this dependency to make it easier to add notes and change thresholds.

A pilot study was initiated to investigate how the framework of the DQD tool might be adapted to interrogate source data. This is an area of interest for OHDSI, EHDEN, and many data partners, and will continue to be researched over the course of the EHDEN project.

4. Conclusion

The Data Quality Dashboard (DQD) developed as part of EHDEN Work Package 4 provides a comprehensive, customizable, and transparent way to both evaluate and communicate the quality of an OMOP CDM instance. We believe the DQD is a strong foundation for data quality reporting, and a key tool to ensure confidence in the evidence generated through the EHDEN federated network. It has proved invaluable during the COVID-19 Rapid Collaboration Call and continues to be adapted to address the needs of the network. The structure of the tool itself is the first of its kind, automating thousands checks based on a small number of check types. This makes it easy to extend and offers endless possibilities on the quest to harmonise fit-for-purpose real world data.

© Copyright 2020 EHDEN Consortium
