# Supplementary material for: Advancing Real-World Evidence Through a Federated Health Data Network (EHDEN): Descriptive Study
Source: J Med Internet Res. 2025 Aug 7;27:e74119. doi: 10.2196/74119 (PMC12331365; doi:10.2196/74119)
Supplement: Multimedia Appendix 3 [file jmir-v27-e74119-s003.pdf]

EHDEN

EUROPEAN HEALTH DATA & EVIDENCE NETWORK

SUB-GRANT AGREEMENT MODEL

For provision of financial support to third parties

Version 3.7

27 August 2019

**This project has received funding from the Innovative Medicines Initiative 2 Joint Undertaking (JU) under grant agreement No 806968.
The JU receives support from the European Union's Horizon 2020 research and innovation programme and EFPIA.**

This EHDEN Sub-Grant Agreement for providing financial support to the Data Partner (hereinafter referred to as the "**Agreement**") is entered into by and between the following parties:

on the one part,

ERASMUS UNIVERSITAIR MEDISCH CENTRUM ROTTERDAM (EMC), established in Dr. Molerwaterplein 40, Rotterdam 3015 GD, Netherlands, with VAT number NL8071811997B01, duly represented for the purposes of signing the Agreement by Dean and Member of the Executive Board, Prof. dr. J.P.T.M. (Hans) van Leeuwen, hereinafter referred as the "**Harmonization Fund Holder**" and acting as Coordinator of the Research and Innovation Action "*European Health Data and Evidence Network*" (hereinafter referred to as "**EHDEN**") and representing the EHDEN consortium members;

and on the other part,

[**ORGANISATION NAME**], established in [Legal Address], with VAT number [VAT_number], duly represented by [Legal Representative], [Legal Representative Position], hereinafter referred to as the **Data Partner**,

Hereinafter individually or collectively referred to as "**Party**" or "**Parties**".

The contracting Parties **HAVE AGREED** to the following terms and conditions including those in the following annexes, which form an integral part of this Sub-Grant Agreement. The Sub-Grant Agreement is composed of:

- Preamble
- Terms and conditions
- **Annex 1**: Workplan
- **Annex 2**: Call Description
- **Annex 3**: Data Partner bank information

A. PREAMBLE

The Innovative Medicines Initiative 2 Joint Undertaking (hereinafter referred as "**IMI**"), and the Harmonization Fund Holder have signed the Grant Agreement no. 806968 for the implementation of the Action "European Health Data and Evidence Network" (hereinafter referred as "**EHDEN**"). The EHDEN Consortium has also signed a Consortium Agreement, further detailing some of the provisions in the Grant Agreement. EHDEN includes specific activities aimed at incentivising the standardisation of health data sources in Europe to the Observational Medical Outcomes Partnership Common Data Model (hereinafter referred as "**OMOP CDM**"). Such activities comprise the launch of periodic EHDEN open calls to interested Data Partners to receive financial support for mapping their data to the OMOP CDM. Such financial support is granted based on transparent, fair and equitable evaluation criteria.

The Data Partner applied for an Open Call organised in the framework of the EHDEN activities as foreseen in the above-mentioned Grant Agreement, and has been selected to receive Financial Support. The application submitted by the Data Partner results in a plan of activities and milestones described in **Annex 1** (hereinafter referred as the "**Work Plan**") that the Data Partner needs to fulfil in order to receive the Financial Support.

The rights and obligations contained in this Agreement are derived from the EHDEN Grant Agreement and Consortium Agreement. The Harmonization Fund Holder is willing to sign this Sub-Grant Agreement on behalf of the EHDEN consortium in compliance with both the EHDEN Grant Agreement and the Consortium Agreement.

The Harmonization Fund Holder is willing to provide Financial Support to the Data Partner for the implementation of the Work Plan, and the Data Partner is willing to receive the Financial Support under the terms and conditions of this Agreement.

The Harmonization Fund Holder is responsible for the execution of this Agreement with the Data Partner. The Data Partner is responsible for the implementation of the work described in the Work Plan.

B. TERMS AND CONDITIONS

1. Subject of the Agreement

1.1. This Agreement sets out the rights and obligations of the Parties as well as the terms and conditions applicable to the provision of the Financial Support awarded to the Data Partner for implementing the Work Plan as set out in **Annex 1** of this Agreement.

1.2. The Agreement specifies the conditions for the implementation of the Work Plan by the Data Partner, within the general framework established in the EHDEN Open Call as described in the Call Description in **Annex 2** of this Agreement.

1.3. The Harmonization Fund Holder receives funding from the Innovative Medicines Agency 2 Joint Undertaking to undertake Open Calls for Grant Awarding to Data Partners. Under the EHDEN Grant Agreement and the Consortium Agreement, some of the obligations of the Harmonization Fund Holder have to be imposed on the Data Partner. Those obligations are reflected in this Agreement.

1.4. The Data Partner acknowledges and agrees that the obligations comprised in this Agreement are fully applicable and shall do everything that is necessary to comply with these obligations, being understood that the Data Partner is bound to this Agreement only and not to the EHDEN Grant Agreement or Consortium Agreement, unless otherwise stated.

2. Financial Support for the Work Plan

2.1. The Data Partner is awarded with **€ xx.xx** (XXXXXX Euro) as financial support ("**Financial Support**") to implement the Work Plan, according to the budget specified in **Annex 1**.

2.2. The Financial Support awarded to the Data Partner is understood to cover all reasonable, actual costs associated to the implementation of the Work Plan. It shall never provide any profit to the Data Partner. The Financial Support is to be considered as a maximum amount and will not be increased in any case, including a situation where the actual costs related to implementing the Work Plan exceed such Financial Support.

2.3. The Data Partner shall manage the Financial Support with due diligence, according to the conditions set out in **Annex 2** to this Agreement, solely for the purposes of implementing the Work Plan and complying with all applicable laws and its own usual policies. The Data Partner shall record the costs associated with implementing the Work Plan in its own accounts, allowing information on such costs to be easily identifiable and available to the Harmonization Fund Holder upon request.

2.4. The awarded Financial Support will be made available to the Data Partner in Euro. No compensation for potential gains or losses due to exchange rates will be considered by either Party. Furthermore it is understood that any taxes (e.g. VAT) are to be considered included in the amount mentioned in section 2.1 and **Annex 1**. The Data Partner is solely responsible in respect of the proper tax treatment of the Financial Support.

3. General obligations to properly implement the Work Plan

3.1. The Data Partner:

a. shall execute the Work Plan in line with the highest quality standards and state-of-the-art scientific and technological development;

b. must implement the Work Plan as described in **Annex 1** and in compliance with the provisions of this Agreement and all legal obligations under applicable EU, international and national law;

c. has full responsibility for implementing the Work Plan properly and in compliance with this Agreement;

d. agrees to be included, without any compensation, in the EHDEN Database Catalogue and EHDEN communication materials intended for the public as an EHDEN "**Data Partner**";

e. agrees to participate in at least one research study promoted by EHDEN, subject to the applicable ethics and other approvals required by the Data Partner's governance structure.

3.2. On behalf of the EHDEN Consortium, the Harmonization Fund Holder shall monitor the implementation of the Work Plan and approve in writing any milestones achieved.

3.3. If the Data Partner does not properly implement the Work Plan, and in particular if it fails to achieve any of the milestones described in **Annex 1**, the Harmonization Fund Holder reserves the right to withhold the corresponding part of the Financial Support until such milestones are achieved and approved.

3.4. Except if the duration of this Agreement is extended pursuant to section 11 below, if the Work Plan cannot be implemented in full for any reason within a maximum period of one year from the date of signature of this Agreement, the Harmonization Fund Holder will reduce the Financial Support and make it correspond only to the milestones that have been achieved and approved, according to the breakdown provided in **Annex 1**. In that event, the Data Partner agrees to return to the Harmonization Fund Holder any amounts that may have been paid in excess of those corresponding to the milestones achieved and approved.

3.5. Parts of the Work Plan will be implemented by an EHDEN-certified small to medium-sized enterprise (hereinafter referred to as "**Certified SME**") as indicated in **Annex 1**, unless otherwise agreed by the EHDEN consortium. For clarity, an agreement of the EHDEN consortium regarding the foregoing needs a decision of the Executive Committee ("**ExCom**"). The procurement procedures that may apply to hire a Certified SME or any other subcontractor to implement the Work Plan will be subject to the corresponding Data Partner's internal policy and usual practice as needed, and will in any case respect all applicable national legislation and fundamental principles such as best value for money.

4. General obligations to inform the Harmonization Fund Holder

4.1. The Data Partner must provide any information requested by the Harmonization Fund Holder in writing at any time to verify the proper implementation of the Work Plan in compliance with the obligations under the Agreement, including information about actual costs incurred.

4.2. The Data Partner must immediately inform the Harmonization Fund Holder of any needed changes in the Work Plan as set out in **Annex 1**. In particular the Data Partner must promptly provide any relevant information on:

a. any circumstances, changes and/or events that may cause deviation from the Work Plan, especially regarding its start and the achievement of the foreseen milestones;

b. any obstacles and/or limitations that may prevent or are likely to affect or delay the Work Plan implementation, including the submission of activity reports to the Harmonization Fund Holder;

d. any relevant changes in the Data Partner legal, financial, technical, organisational or ownership status that may affect implementation of the Work Plan;

e. any circumstances that may affect the Data Partner's due diligence in the management of the Financial Support or the compliance with the obligations of this Agreement.

4.3. The Parties shall only be able to modify the Work Plan included as **Annex 1** by mutual agreement.

5. Final report

5.1 The Data Partner shall provide the Harmonization Fund Holder with a final report in accordance with the timing and conditions set out in this Agreement.

5.2 The final report must comprise the following documents:

a. Description of activities carried out according to the Work Plan;

b. Financial cost statement including details on the costs incurred in during the implementation of the Work Plan;

c. Brief summary including explanations justifying any differences and deviations between the Work Plan as set in **Annex 1** and the work actually carried out (if any);

d. Request for final payment.

5.3 The financial cost statement will be expressed in EURO (€). All costs issued in another currency, must be converted into EURO (€) for the purposes of reporting.

5.4. The Data Partner must submit the final report within 30 days following the end of the Work Plan.

6. Payments

6.1. The Financial Support indicated in provision 2.1 of this Agreement will be paid by the Harmonization Fund Holder to the Data Partner in a series of installments as described in **Annex 1**. Payments will be made upon achievement and approval of the corresponding milestones indicated in **Annex 1**.

6.2. Notwithstanding provision 6.1, an advance payment corresponding to 20% of the total Financial Support can be requested by the Data partner upon signature of this Agreement. In case such advance payment is requested, this advance payment will have the sole purpose of facilitating the start of activities for the Data Partner and will be offset from the rest of payments foreseen in **Annex 1** proportionately.

6.3. All payments made by the Harmonization Fund Holder to the Data Partner shall never cumulatively exceed the Financial Support indicated in provision 2.1 of this Agreement, and shall never exceed the total costs reported by the Data Partner in its final report.

6.4. A final payment shall be made upon completion of the Work Plan and approval by the Harmonization Fund Holder of the final report described in section 5 of this Agreement. The final payment will cover any outstanding part of the Financial Support that has not yet been paid, provided that the Data Partner reports total costs equal or higher than the Financial Support. Otherwise, the final payment will cover only any unpaid amounts up to the total costs reported by the Data Partner. In the event that the costs reported by the Data Partner are lower than the amount already paid, the Data Partner shall return the excess payment to the Harmonization Fund Holder without undue delay.

6.5. Payments by the Harmonization Fund Holder are considered to have been carried out on the date when they are debited to the Data Partner's bank account.

6.6. Payments will be made by the Harmonization Fund Holder by bank transfer to the account indicated in **Annex 3** to this Agreement. The Data Partner shall notify the Harmonization Fund Holder of any changes in the information contained in **Annex 3** as soon as possible. The Harmonization Fund Holder shall not in any case be liable for any late payment derived from a change in the financial information of the Data Partner.

6.7. The Harmonization Fund Holder shall bear the cost of transfers charged by its bank. The Data Partner shall bear the cost of transfers charged by its bank. The Party causing a repetition of a transfer shall bear the cost of the repeated transfer.

7. Consequences of non-compliance

7.1 If the Data Partner fails to comply with any of the obligations set in this Agreement, the Harmonization Fund Holder may suspend any payment and require additional information for clarification.

7.2 If the Data Partner fails to comply with their obligations within one month following a written request for clarification, the Harmonization Fund Holder may terminate the Agreement.

8. Confidentiality

8.1 During implementation of the Work Plan and for **four (4) years** after the period of 66 months as of 1 November 2018, the EHDEN consortium members and the Data Partner must keep confidential any data, documents or other material (in any form) that is identified as confidential at the time it is disclosed ("**Confidential Information**"). If information has been identified as confidential only orally, it will be considered to be confidential only if this is confirmed in writing within 15 days of the oral disclosure. Unless otherwise agreed between the Data Partner and the EHDEN consortium, they may use Confidential Information only to implement the Work Plan.

8.2 The Data Partner may ask the Harmonization Fund Holder to request to the IMI2 JU to keep such information confidential for an additional period beyond the initial four years.

8.3 The Data Partner may disclose confidential information to their personnel, or third parties involved in the action only if they:
(a) need to know to implement the Work Plan, and
(b) are bound by an obligation of confidentiality.

8.4 The confidentiality obligations mentioned in this article no longer apply if:
(a) the disclosing Party agrees to release the other Party;
(b) the information was already known by the recipient or is given to him without obligation of confidentiality by a third party that was not bound by any obligation of confidentiality;
(c) the recipient proves that the information was developed without the use of confidential information;
(d) the information becomes generally and publicly available, without breaching any confidentiality obligation, I(e) the disclosure of the information is required by EU or national law.

8.5 It is understood by the Data Partner that the IMI2 JU may disclose Confidential Information to its staff, other EU institutions and bodies. It may disclose Confidential Information to third parties, if:
(a) this is necessary to implement the EHDEN Grant Agreement or safeguard the EU's or IMI2 JU's financial interests, and
(b) the recipients of the information are bound by an obligation of confidentiality.

8.6 The consequences of non-compliance as mentioned in article 7 of this Agreement ('*Consequences of non-compliance*') are applicable accordingly.

9. Visibility of EHDEN

9.1 The Data Partner declares their willingness to support EHDEN by attending its public events on invitation and by presenting the results of the Work Plan.

9.2 When promoting the Work Plan and its major results, the Data Partner shall display the EHDEN logo, the IMI2 JU and EFPIA logos, and the EU emblem and include the following text: "This project has received funding from the Innovative Medicines Initiative 2 Joint Undertaking (JU) under grant agreement no 806968. The JU receives support from the European Union's Horizon 2020 research and innovation programme and EFPIA".

9.3 The Data Partner authorizes the Harmonization Fund Holder to use the Data Partner's logo and name for all purposes related to communication of EHDEN activities.

10. Liability

10.1 Each Party represents and warrants that (i) it has the full right and authority to enter into and perform its obligations as set forth in this Agreement; (ii) the execution, delivery and performance of this Agreement have been duly authorized by all necessary corporate action on its part; (iii) it will not grant any rights in conflict with any of the rights granted in this Agreement; (iv) the rights granted pursuant to this Agreement do not and will not infringe with any Third Party rights and, notwithstanding the foregoing, it will notify the other Parties promptly after becoming aware of any Third Party right infringement.

10.2 The Data Partner agrees that the IMI2 JU cannot be held liable for any damage caused to and/or by the Data Partner as a consequence of implementing any action in the framework of the EHDEN Project.

11. Term and Termination

11.1. Unless terminated earlier in accordance with the provisions of this Agreement, the term of this Agreement shall commence on the date of its full signature and shall remain in effect until the completion of the Work Plan, with a maximum duration of 12 months, unless this period is extended by mutual agreement of the Parties in writing.

11.2. The Agreement is automatically terminated at the end of the EHDEN Project.

11.3. Termination for Breach. Either party may terminate this Agreement if the other party materially breaches a provision of this Agreement and fails to cure such breach within thirty (30) days of receipt of written notice describing the breach in reasonable detail.

12. Conflict of Interest

12.1 The Data Partner agrees to take all measures to prevent any situation where the impartial and objective implementation of the action is compromised for reasons involving economic interest, political or national affinity, family or emotional ties or any other shared interest ("**Conflict of Interests**").

12.2 The Data Partner must formally notify to the Harmonization Fund Holder without delay of any situation constituting or likely to lead to a Conflict of Interests and immediately take all the necessary steps to rectify this situation.

12.3 The Harmonization Fund Holder may verify that the measures taken are appropriate and may require additional measures to be taken by a specified deadline.

12.4 The consequences of non-compliance as mentioned in article 7 of this Agreement ('*Consequences of non-compliance*') are applicable accordingly.

13. Miscellaneous

13.1 The Data Partner undertakes to comply with all applicable laws, rules, regulations, ordinances, and directives. In addition to the foregoing, the Data Partner acknowledges that this Agreement is a derivative of the EHDEN Grant Agreement. The Data Partner explicitly agrees to comply with the EHDEN Grant Agreement as if it is a Beneficiary, to the extent its specific applicable articles are mentioned in this Sub Grant Agreement.

13.2 The Data Partner agrees to ensure that the IMI2 JU, the European Commission, the European Court of Auditors (ECA) and the European Anti-Fraud Office (OLAF) can exercise their rights of Checks, Reviews and Audits as well as Evaluation of the Impact if necessary in respect of the EHDEN project, as established in the EHDEN Grant Agreement.

14. Governing law

14.1. This Agreement shall be governed by and interpreted in accordance with the law of the Netherlands.
14.2. Any dispute which cannot be settled amicably shall be finally settled by the court of Rotterdam, the Netherlands.

<<< SIGNATURES WILL FOLLOW ON THE NEXT PAGE >>>

IN WITNESS WHEREOF, the Parties have executed this Agreement in duplicate originals by their duly authorized officers or representatives,

On behalf of the EHDEN consortium:

ERASMUS UNIVERSITAIR MEDISCH CENTRUM ROTTERDAM

………………

Name: Prof. dr. J.P.T.M. (Hans) van Leeuwen

Title: Dean and Member of the Executive Board

and

As Data Partner:

[NAME]

……………….

Name:

Title:

ANNEX 1. WORKPLAN

[NOTE: This Annex will include the activities that will be performed, and the associated resources, milestones and payments. The contents below is only indicative, based on a default model workplan. It will be adjusted as necessary on a case by case basis before signature.]

Data set(s) identifier(s):
[unique identifier for each dataset that will be mapped to the OMOP CDM]

Data set(s) description:
[description of the datasets, scope and size – will need to be aligned with the description provided in the application form]

Activities:
[detailed description of the activities that will be undertaken leading to milestone completion, including any procurement arrangements or preparatory work needed, responsible, timelines]

Milestones/payments:

Milestone	***Grant Percentage***	***Estimated Timelines***
Shared ETL documentation	*30%*	*Month 3*
ETL Implemented and Infrastructure Operational	*40%*	*Month 6*
Database catalogue entry following final inspection by certified SME, final report	*30%*	*Month 7*

Estimated costs (in Euro):
[estimation of resources needed to fulfil the activities described above, e.g.:

- *Personnel costs*
- *Other direct costs (e.g. consumables)*
- *Subcontracting/external services (e.g. to certified SME for mapping/inspection services)*
- *Total costs:*

(cost categories are indicative)]

ANNEX 2. CALL DESCRIPTION

Currently available on www.ehden.eu and added in sub-grant agreement upon signature.

ANNEX 3. DATA PARTNER BANK INFORMATION

FINANCIAL IDENTIFICATION

Please use CAPITAL LETTERS and LATIN CHARACTERS when filling in the form.

BANKING DETAILS ①			
ACCOUNT NAME ②			
IBAN/ACCOUNT NUMBER ③			
CURRENCY			
BIC/SWIFT CODE		BRANCH CODE ④	
BANK NAME			
ADDRESS OF BANK BRANCH			
STREET & NUMBER			
TOWN/CITY		POSTCODE	
COUNTRY			

ACCOUNT HOLDER'S DATA (AS DECLARED TO THE BANK)			
ACCOUNT HOLDER			
STREET & NUMBER			
TOWN/CITY		POSTCODE	
COUNTRY			

REMARK

BANK STAMP + SIGNATURE OF BANK REPRESENTATIVE ⑤	DATE (Obligatory)
	SIGNATURE OF ACCOUNT HOLDER (Obligatory)

① Enter the final bank data and not the data of the intermediary bank.

② This does not refer to the type of account. The account name is usually the one of the account holder. However, the account holder may have chosen to give a different name to its bank account.

③ Fill in the IBAN Code (International Bank Account Number) if it exists in the country where your bank is established

④ Only applicable for US (ABA code), for AU/NZ (BSB code) and for CA (Transit code). Does not apply for other countries.

⑤ It is preferable to attach a copy of RECENT bank statement. Please note that the bank statement has to confirm all the information listed above under 'ACCOUNT NAME', 'ACCOUNT NUMBER/IBAN' and 'BANK NAME'. With an attached statement, the stamp of the bank and the signature of the bank's representative are not required. The signature of the account-holder and the date are ALWAYS mandatory.
